# Advanced nanocatalytic medicine for genitourinary diseases: Reactive oxygen modulation, precision therapeutics, and clinical translation

**DOI:** 10.1016/j.mtbio.2026.103572

**Published:** 2026-08-14

**Authors:** Huimin Li, Ye Geng, Lina Wang, Shangxu Jiang, Ming Du, Yanan Sun, Li Li, Xinwang Zhu

**Affiliations:** a Department of Nephrology, The Fourth Affiliated Hospital of China Medical University, Shenyang, Liaoning, 110032, China; b Department of Blood Purification Room, The First Hospital of China Medical University, Shenyang, Liaoning, 110001, China; c Department of Central Sterile Supply Department, The First Hospital of China Medical University, Shenyang, Liaoning, 110001, China; d Australian Institute for Bioengineering and Nanotechnology, The University of Queensland, St Lucia, QLD, 4072, Australia; e Department of Nuclear Medicine, Shengjing Hospital of China Medical University, Shenyang, Liaoning, 110004, China; f Department of Nephrology, The First Hospital of China Medical University, Shenyang, Liaoning, 110000, China; g Gulbali Research Institute, Charles Sturt University, Wagga Wagga, NSW, 2678, Australia

**Keywords:** Genitourinary malignancies, Nanocatalytic medicine, Biocatalytic nanomaterials, Urological diseases, Precision therapeutics, Ferroptosis regulation

## Abstract

Urological, kidney, and reproductive diseases comprise heterogeneous disorders, both malignant and nonmalignant, characterized by oxidative stress, inflammation, metabolic dysfunction, fibrosis, immune dysregulation, and treatment resistance. Advanced nanocatalytic medicine (NCM) offers a precise therapeutic approach by exploiting disease-associated biochemical abnormalities, including acidic pH, hypoxia, elevated hydrogen peroxide (H_2_O_2_) levels, altered glutathione (GSH) metabolism, mitochondrial dysfunction, and redox imbalance. Biocatalytic nanomaterials, including nanozymes, metallic and metal-oxide nanostructures, single-atom catalysts, hybrid platforms, biomimetic systems, and stimuli-responsive materials, enable context-dependent regulation of reactive oxygen species (ROS). Nanocatalytic platforms exploit these conserved tumor microenvironmental characteristics across ovarian, endometrial, cervical, bladder, renal cell, prostate, and upper tract urothelial cancers through Fenton/Fenton-like catalysis, ROS amplification, GSH depletion, ferroptosis induction, immune microenvironment remodeling, biomimetic targeted delivery, and image-guided precision therapy. Beyond oncology, antioxidant nanozymes have also shown therapeutic potential in selected non-malignant renal and reproductive disorders through ROS scavenging and redox restoration. These conserved mechanisms support chemodynamic therapy (CDT), catalytic phototherapy, ferroptosis regulation, metabolic modulation, immunocatalytic remodeling, biomimetic targeting, targeted drug delivery, and multimodal image-guided precision therapy for diverse genitourinary (GU) malignancies. Artificial intelligence (AI)-assisted nanozyme engineering, multi-omics stratification, organoid-guided screening, and computational modeling may accelerate personalized treatment; however, biosafety, pharmacokinetics, manufacturing, and regulatory challenges remain unresolved. Overall, the shared biochemical vulnerabilities of GU malignancies provide a unified framework for translating NCM into disease-specific precision oncology, while also offering opportunities for the treatment of selected non-malignant disorders.

## Graphical abstract

**Figure:**
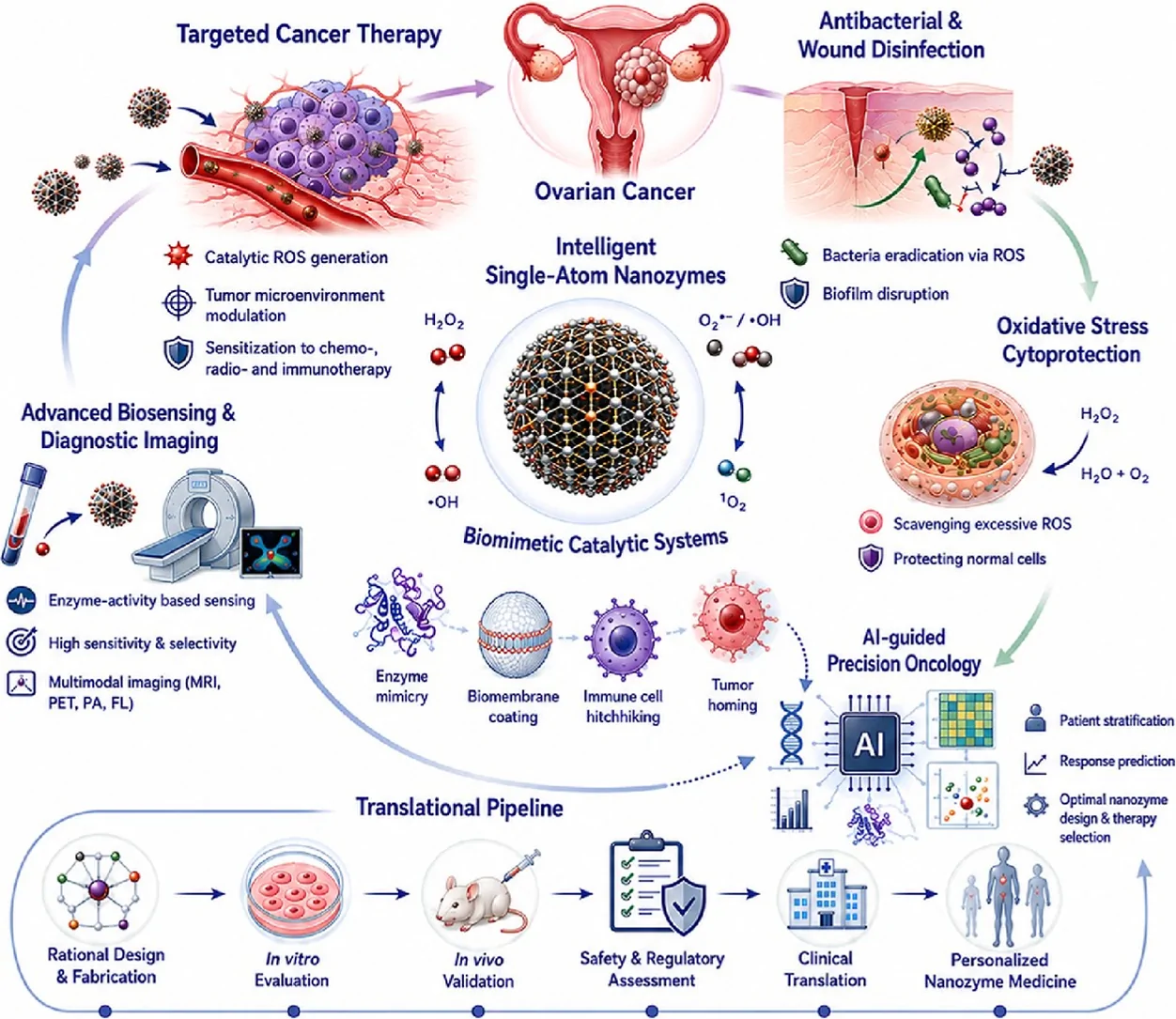

## 1 Introduction

Urological and reproductive diseases include a wide range of malignant, inflammatory, metabolic, infectious, ischemic, and degenerative diseases of the kidneys, urinary tract, and male and female reproductive organs. These diseases include ovarian, cervical, endometrial, prostate, bladder, and kidney cancers; conditions associated with fertility; inflammatory diseases of the reproductive organs; urinary and kidney dysfunction; and chronic kidney diseases. This clinical burden is exacerbated by the fact that these diseases are often underdiagnosed, involve highly variable mechanisms, recur or progress over time, and remain difficult to target therapeutically [[1], [2], [3]].

Nephritis is an immune-mediated or inflammatory process that injures the renal tissue. Nephrotic syndrome is characterized by the disruption of the glomerular filtration barrier, resulting in increased proteinuria, hypoalbuminemia, edema, and an increased risk of thrombotic and infectious complications. Acute kidney injury may be caused by ischemia-reperfusion injury, sepsis, nephrotoxic drugs, oxidative stress, and systemic inflammation, and is characterized by a rapid decrease in renal function. Although the underlying causes of these disorders differ, they commonly exhibit overlapping pathological hallmarks, including increased ROS production, mitochondrial dysfunction, endothelial injury, infiltration of immune cells, production of inflammatory cytokines, injury to tubules or glomeruli, and progressive fibrosis.

Reproductive and urological cancers pose further therapeutic complications due to tumor heterogeneity, metastatic spread, an immunosuppressive environment, metabolic adaptation, redox regulation, chemotherapy resistance, and resistance to targeted therapies. Standard treatments, such as surgery, chemotherapy, radiotherapy, immunotherapy, hormonal therapy, corticosteroids, immunosuppressive agents, and supportive renal care, are effective in managing the disease. However, these treatments are limited by systemic toxicity, poor tissue specificity, suboptimal drug accumulation, disease recurrence, treatment resistance, and the inability to completely reverse organ damage or fibrosis [[4], [5], [6]].

Although ovarian cancer has historically been the predominant focus of nanocatalytic research, many of the pathological characteristics exploited by catalytic nanomedicine are conserved across the broader spectrum of GU malignancies, including endometrial and cervical cancers, bladder cancer, renal cell carcinoma (RCC), prostate cancer, and upper tract urothelial carcinoma. These malignancies commonly exhibit acidic tumor microenvironments, elevated endogenous H_2_O_2_ levels, increased GSH-dependent antioxidant capacity, hypoxia, metabolic reprogramming, immunosuppressive cellular networks, and therapeutic resistance, providing common biochemical substrates for Fenton/Fenton-like catalysis, ROS amplification, ferroptosis induction, immune remodeling, and biomimetic targeted delivery systems. Consequently, nanocatalytic strategies initially developed for ovarian cancer possess broad mechanistic relevance and translational potential across the full spectrum of GU malignancies, although disease-specific molecular targets, patterns of metastatic dissemination, and standard treatment paradigms require tailored therapeutic optimization.

Importantly, the redox intervention required varies across different disease contexts. Catalytic ROS amplification may be beneficial for killing malignant or infected cells. In contrast, most other inflammatory conditions, such as nephritis, nephrotic syndrome, and ischemic kidney injury, require catalytic ROS scavenging and antioxidant regulation in renal and reproductive tissues. Therefore, precision NCMs must be engineered to control ROS production or elimination, inflammatory signaling, oxygen homeostasis, metabolism, and immune responses in a disease-specific manner. The use of stimuli-responsive nanoplatforms that alter their catalytic activity in response to local pathological conditions and biocatalytic nanozymes may open new opportunities for the targeted and individualized treatment of urological, renal, and reproductive diseases [[7], [8], [9]].

NCM has emerged as an advanced therapeutic strategy that employs catalytic nanomaterials to accentuate biochemical reactions in tumor tissues and boost anti-cancer activity. In contrast to conventional nanotherapeutics with passive target, nanozymes and other catalytic nanoplatforms can actively interact in the treatment process via enzyme-like reactions, substrate conversion, ROS regulation, oxygen generation, antioxidant depletion, and modulation of inflammatory and metabolic pathways [10,11]. They can be designed to possess specific physicochemical properties and catalytic activities that target pathological microenvironments, such as acidic pH, hypoxia, elevated H_2_O_2_ levels, abnormal immune signaling, mitochondrial dysfunction, and altered GSH concentrations, and provide disease-responsive therapeutic interventions, as reported by Hu and Zhang [12,13].

The therapeutic effect of NCM is disease-specific. Catalytic biomaterials can induce oxidative stress in cells, metabolic stress in tumors, ferroptosis, and apoptosis, and enhance the efficacy of chemotherapy, phototherapy, and immunotherapy in urological and reproductive cancers. In non-malignant renal and reproductive diseases, such as nephritis, nephrotic syndrome, acute kidney injury, ischemia-reperfusion injury, or inflammatory reproductive diseases, the administration of nanozymes containing superoxide dismutase (SOD), catalase (CAT), glutathione peroxidase (GP_x_), or peroxidase-like activity can be oriented towards the elimination of excess ROS, inhibition of inflammatory cascades, stabilization of mitochondrial functions, and prevention of tissue fibrosis. Therefore, NCM involves cytotoxic or pro-oxidant strategies for the selective destruction of pathological cells, as well as antioxidant strategies for the protection and regeneration of injured tissues [14].

Combination of catalytic nanotechnology and urology/nephrology/reproductive medicine has led to the development of multifunctional platforms that can perform simultaneous diagnosis, delivery, molecular imaging, and therapeutic activation. Stimuli-responsive and biomimetic nanozymes have the potential to enhance the accumulation of drugs in the target tissues, control the catalytic activity in response to local pathological signals, and minimize systemic toxicity. These systems also enable synergies with other modalities, such as CDT, catalytic phototherapy, metabolic therapy, ferroptosis induction, anti-inflammatory immunocatalytic therapy, and imaging-guided theranostics [[15], [16], [17]].

Rapidly emerging therapeutic platforms, such as biocatalytic nanomaterials, mimic natural enzymatic activity and control redox processes in diseased tissues. Dysregulated ROS homeostasis, mitochondrial dysfunction, hypoxia, metabolic reprogramming, abnormal immune activation, and inadequate antioxidant defense are frequently reported to be involved in urological and reproductive diseases, such as renal and reproductive malignancies, nephritis and nephrotic syndrome, acute kidney injury, ischemia-reperfusion injury, and inflammatory reproductive diseases. However, the effects of these abnormalities on the biological aspects of the disease vary according to the disease type [18,19]. In malignant cells, selective amplification of ROS can lead to oxidative damage and death of tumor cells, whereas in inflammatory and degenerative diseases, uncontrolled ROS production must be neutralized to maintain tissue integrity and organ function, as shown by Singh [20].

Hence, biocatalytic nanomaterials can be designed to exhibit pro-oxidant or antioxidant catalytic activities. Peroxidase-, oxidase-, and Fenton/Fenton-like catalytic systems can exploit the acidic pH and increased levels of H_2_O_2_, hypoxia, and GSH overexpression in urological and reproductive cancers to generate high levels of ROS, deplete antioxidant levels, and disrupt mitochondrial metabolism, ultimately inducing apoptosis, ferroptosis, and immunogenic cell death (ICD). These catalytic mechanisms can increase tumor selectivity, overcome therapeutic resistance, and enhance sensitivity to chemotherapy, phototherapy, and immunotherapy [21,22].

In addition, disease-associated signals, such as low pH, hypoxia, elevated ROS, enzyme dysfunction, and metabolic dysfunction, can make the functions of nanoplatforms disease-responsive, further increasing therapeutic precision. These can target catalysis to diseased tissues, increase the therapeutic effect, and decrease the off-target toxicity. Drug delivery, immunomodulation, metabolic regulation, regenerative therapy, molecular imaging, and theranostic monitoring are other potential applications of biocatalytic nanomaterials. Thus, the bidirectional and disease-specific regulation of ROS is a key concept in NCM for urological and reproductive diseases [9,11,23].

This review summarizes recent progress in biocatalytic nanozymes and ROS-modulating nanocatalytic systems in urological and reproductive diseases, such as cancer, nephritis, nephrotic syndrome, and kidney injury. Additionally, it discusses AI-aided design, precision targeting, theranostics, biosafety, pharmacokinetics, manufacturing, and clinical translation in nanomedicine.

## 2 Biological and catalytic basis of NCM in urological, kidney, and reproductive diseases

Nanocatalytic therapeutics haven shown great potentials in ovarian cancer treatment. Recent studies have also demonstrated that the similar catalytic principles are broadly applicable to other GU malignancies, including endometrial and cervical cancers, bladder cancer, RCC, prostate cancer, and upper tract urothelial carcinoma. Despite their distinct tissue origins, these malignancies exhibit common redox-associated vulnerabilities, including elevated H_2_O_2_ levels, enhanced GSH-dependent antioxidant defenses, hypoxia, extracellular acidosis, metabolic rewiring, and immune suppression. Accordingly, the mechanistic concepts discussed in this section as broadly relevant to GU malignancies unless otherwise specified.

### 2.1 Oxidative stress and redox biology

**Fig. 1:**
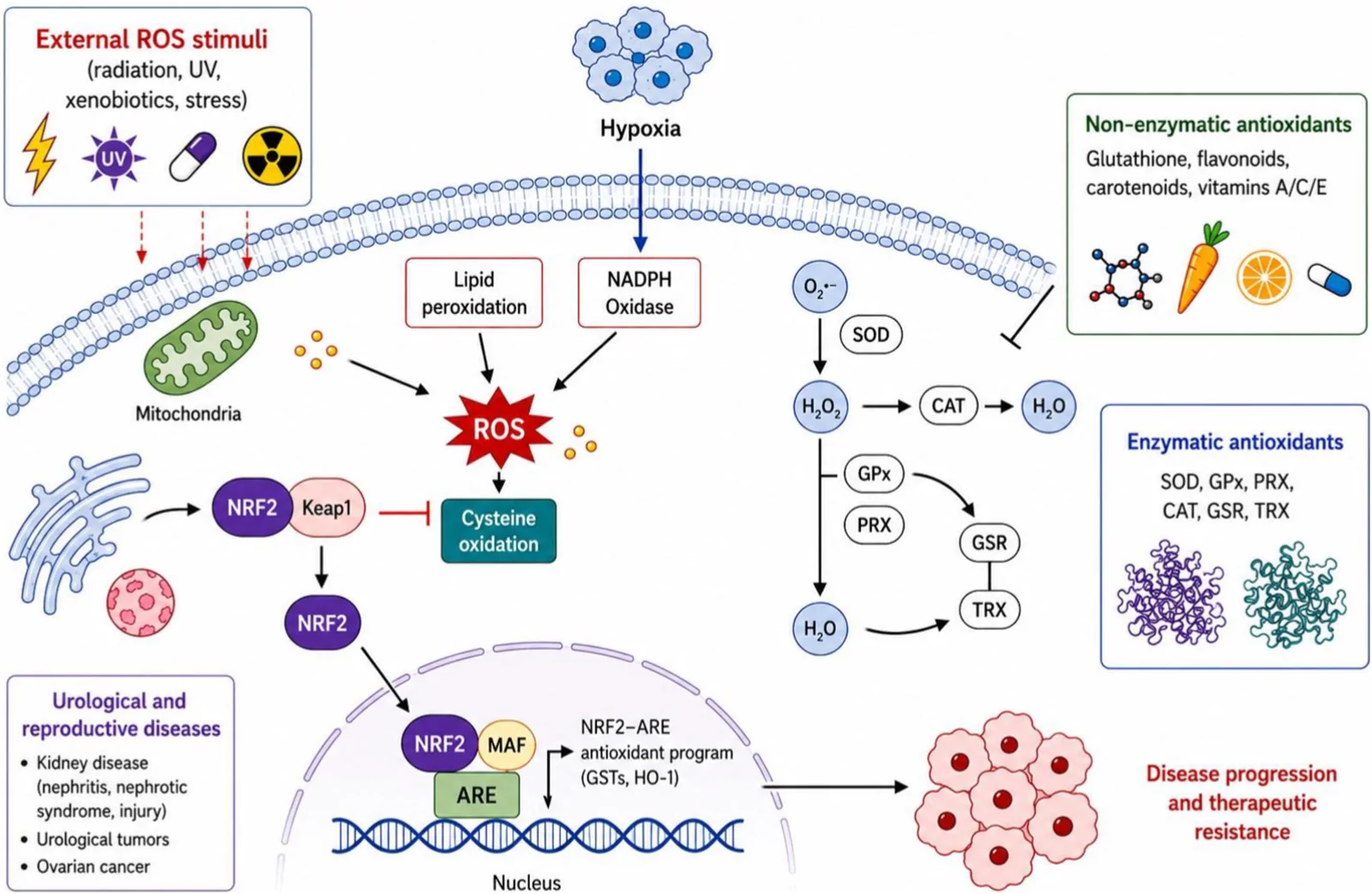
Redox imbalance and NRF2-mediated antioxidant defense in urological and reproductive diseases. External stimuli, hypoxia, mitochondrial dysfunction, lipid peroxidation, and NADPH oxidase increase ROS production, activating the KEAP1-NRF2-ARE pathway and enzymatic and non-enzymatic antioxidant systems. Persistent oxidative stress promotes disease progression and therapeutic resistance in patients with cancer. Image created with BioRender; concept adapted from previous studies [28,29].

Redox-sensitive signaling pathways, particularly those involving ROS, are involved in the initiation, progression, and therapeutic responses of urological and reproductive diseases. These dysregulated processes can produce chronic oxidative imbalance in renal, urinary, and reproductive tissues through mitochondrial dysfunction, tissue hypoxia, metabolic reprogramming, lipid peroxidation, and NADPH oxidase [24,25]. High ROS levels in urologic and reproductive cancers can lead to genomic instability, proliferation, metastasis, immune evasion, and resistance to therapy. In contrast, in nephritis, nephrotic syndrome, acute kidney injury, ischemia-reperfusion injury, and inflammatory reproductive disorders, excess ROS impairs endothelial, podocyte, and tubular functions, damages mitochondria, activates inflammatory pathways, and promotes fibrosis. Oxidative damage can be further magnified by external stressors such as radiation, xenobiotics, nephrotoxic drugs, infections, and cellular stress. The molecular interactions between ROS generation, antioxidant defense, inflammation, tissue damage, and therapeutic resistance are shown in Fig. 1 [26,27].

Diseased tissues protect themselves from oxidative effects via enzymatic and non-enzymatic mechanisms. Enzymatic antioxidants, including SOD, CAT, GPx, peroxiredoxin (PRX), GSH reductase (GSR), and thioredoxin (TRX), regulate the detoxification of superoxide and H_2_O_2_ and maintain cellular redox states. Non-enzymatic antioxidants include GSH, flavonoids, carotenoids, and vitamins A, C, and E, which offer protection against oxidative stress. The dissociation of *NRF2* from Keap1 and activation of the *NRF2*–ARE pathway promote the transcription of antioxidant genes, including *GSTs* and *HO-1*, which contribute to increasing cellular resistance to oxidative stress and chemoresistance. In damaged renal and reproductive tissues, the activation of these pathways is vital for the regulation of antioxidant responses, thus allowing a reduction in inflammation and the maintenance of tissue function [[30], [31], [32]].

Even with these protective actions, excessive ROS production that exceeds cellular tolerance levels leads to mitochondrial dysfunction, DNA fragmentation, membrane lipid oxidation, and ferroptotic cell death. Different therapeutic opportunities are provided by this redox vulnerability in different disease contexts. Nanozyme engineering has been proposed to improve ROS production, deplete intracellular GSH, inhibit GSH synthesis, and increase lipid peroxidation in urological and reproductive cancers to selectively target malignant cells. In contrast, nephritis, nephrotic syndrome, kidney injury, and inflammatory reproductive disorders have been investigated as potential targets for treatment with ROS-scavenging nanozymes, which provide antioxidant, mitochondrial protective, and antifibrotic effects. Hence, catalytic ROS amplification, antioxidant restoration, oxygen regulation, GSH modulation, and ferroptosis control are significant disease-adapted strategies for precision nanocatalytic therapy (Table 1) [19,33,34].

**Table 1:** Major catalytic mechanisms, ROS modulation strategies, biological targets, representative GU malignancies, therapeutic outcomes, and key references.

Catalytic nanomaterial/system	Catalytic mechanism	ROS/redox-modulation strategy	Biological target/process	Therapeutic outcome	Ref
Iron oxide nanozymes	Fenton reaction	Hydroxyl radical generation	Redox imbalance in urological and reproductive tumors	Apoptosis, ferroptosis, and enhanced tumor cytotoxicity	[21]
Copper-based nanozymes	Cu^+^/Cu^2+^ redox cycling	ROS amplification and GSH depletion	Tumor antioxidant defense and mitochondrial function	Enhanced catalytic tumor destruction	[35,36]
Manganese dioxide nanozymes	CAT-like activity	H_2_O_2_ decomposition and oxygen generation	Hypoxia in tumors and ischemic tissues	Improved therapeutic sensitivity	[37,38]
Cerium oxide nanozymes	Redox cycling	ROS scavenging and redox restoration	Oxidative stress in nephritis, nephrotic syndrome, and kidney injury	Reduced inflammation and renal tissue damage	[13,39]
SOD/CAT-mimicking nanozymes	Antioxidant enzyme mimicry	Superoxide and H_2_O_2_ elimination	Mitochondrial and tubular oxidative injury	Protection against acute kidney injury and fibrosis	[19,40]
Platinum nanozymes	Oxidase-, peroxidase-, or CAT-like activity	Context-dependent ROS generation or scavenging	Mitochondrial dysfunction and redox imbalance	Tumor cytotoxicity or protection of injured tissues	[41,42]
Gold-based catalytic nanoparticles	Photothermal catalytic amplification	ROS and thermal-stress generation	Tumor metabolism and cellular membranes	Synergistic tumor destruction	[43,44]
Single-atom nanozymes	Atomically dispersed catalysis	Efficient ROS conversion	Disease-specific catalytic pathways	Precision therapeutic activation	[45,46]
GOx-loaded nanozymes	Glucose catalytic oxidation	H_2_O_2_ generation and ATP depletion	Tumor metabolism	Starvation-enhanced catalytic therapy	[46,47]
Renal-targeted antioxidant nanozymes	ROS-scavenging catalysis	Suppression of oxidative and inflammatory stress	Glomerular, podocyte, and tubular injury	Renoprotection in nephritis, nephrotic syndrome, and kidney injury	[19,48]
Biomimetic membrane-coated nanozymes	Tissue-homing catalytic delivery	Localized ROS amplification or scavenging	TME	Improved accumulation and reduced off-target toxicity	[49,50]
Exosome-inspired catalytic systems	Biohybrid catalytic delivery	Intracellular redox and inflammatory modulation	Cellular communication and tissue-repair pathways	Enhanced penetration, biocompatibility, and therapeutic delivery	[51,52]
pH-responsive catalytic nanoplatforms	Acidic-microenvironment activation	Localized ROS generation or drug release	Acidic tumors and inflammatory lesions	Disease-selective catalytic therapy	[53,54]
Hypoxia-responsive nanozymes	Oxygen-regulated catalysis	Hypoxia alleviation and ROS modulation	Hypoxic tumors and ischemic kidney or reproductive tissues	Relief hypoxia	[55,56]
Ferroptosis-inducing nanozymes	Lipid-peroxidation regulation	Ferroptosis induction or suppression	Tumors or oxidatively injured renal cells	Tumor elimination or prevention of renal ferroptotic injury	[57,58]
Immunocatalytic nanozymes	ROS-mediated immune regulation	Immunogenic ROS amplification or inflammatory ROS suppression	Dendritic cells, T cells, macrophages, and inflammatory pathways	Antitumor immunity or attenuation of pathological inflammation	[59,60]
Theranostic catalytic nanoplatforms	Integrated imaging and catalysis	Real-time monitoring of ROS and catalytic activity	Precision urology, nephrology, and reproductive medicine	Imaging-guided and personalized therapy	[61,62]

### 2.2 Pathological microenvironments and catalytic therapeutic opportunities

In urological and reproductive diseases, there are multiple possibilities for selective catalytic activation to take place. Alterations in oxygenation, extracellular acidity, H_2_O_2_ concentration, GSH status, inflammatory mediators, and metabolism are common features of these pathological microenvironments [63]. Acidic environments can promote catalytic ion release and accelerate Fenton or Fenton-like reactions in malignant tissues. Under the influence of kidney diseases, ischemia, inflammation, toxic effects, and disturbance of microcirculation favor the accumulation of ROS, causing mitochondrial dysfunction, tubular or glomerular damage, and fibrotic remodeling of the kidney tissue [64,65].

Urological and reproductive cancers are known to have immunosuppressive microenvironments that include tumor-associated macrophages (TAMs), regulatory T-cells, myeloid-derived suppressor cells, and cytokines. In contrast, nephritis and acute kidney injury are typically associated with excessive activation of neutrophils, macrophages, complement pathways, inflammatory cytokines, and adaptive immune responses. Thus, catalytic nanomedicine can be engineered to trigger immunogenic oxidative stress in tumor cells or to lower pathological ROS and/or inflammation in non-cancerous tissues. ROS generation, amplification, depletion, and scavenging, along with mitochondrial protection and immune modulation, can be tailored to each disease. The ability of endogenous H_2_O_2_ and metabolic abnormalities to act as catalytic substrates or activation signals allows for local therapeutic effects without a general systemic effect, which could be dangerous [[66], [67], [68]].

Responsive nanoplatforms enable multifunctional catalytic systems that combine CDT, ferroptosis regulation, immunocatalytic therapy, antioxidant therapy, and tissue protection. Therapeutic precision and biosafety will be improved by smart catalytic materials that are sensitive to hypoxia, acidic pH, oxidative stress, inflammation, and metabolic abnormalities. These systems are involved in tumor-selective catalytic amplification and antioxidant depletion in cancer. They have been shown to be protective against oxidative injury, inflammatory signals, and fibrosis and to promote tissue repair in nephritis, nephrotic syndrome, kidney injury, and inflammatory reproductive diseases. Therefore, pathological microenvironment-responsive catalysis offers a general platform for the precision treatment of diseases related to the urological, renal, and reproductive systems (Table 1) [61,69,70].

### 2.3 Principles of catalytic nanotherapeutics

Catalytic nanotherapeutics use engineered nanomaterials to trigger, control, and catalyze biochemical reactions in diseased tissues. Fenton and Fenton-like reactions allow transition-metal ions to convert endogenous H_2_O_2_ into highly toxic hydroxyl radicals, thus creating a high level of oxidative stress in malignant urological and reproductive tissues. Iron, copper, manganese, and cobalt nanozymes possess tunable catalytic activities and the ability to selectively amplify ROS in tumors. These reactions disrupt mitochondrial function, redox homeostasis, and cellular metabolism, eventually causing apoptosis, ferroptosis, and ICD [22,71,72]. Catalytic nanomaterials are engineered to remove ROS, break down H_2_O_2_, and inhibit oxidative inflammatory pathways in nonmalignant diseases.

Enzyme-mimicking nanozymes have oxidase, peroxidase, CAT, SOD, or GPx-like activity, which can regulate ROS metabolism in both directions and regulate oxygen metabolism. Pro-oxidant systems have been shown to either produce ROS or deplete GSH, whereas antioxidant systems scavenge superoxide and H_2_O_2_ restore redox balance, and protect renal and reproductive tissues. Tumors can have a higher production of ROS, depletion of GSH, and enhanced lipid peroxidation, damaging the tumor cells. This enhances the therapeutic efficiency and responsiveness of the treatment by cascading catalytic reactions and linking multiple enzyme-like activities. Another approach is to use orthogonal catalytic systems that activate therapeutic reactions without causing significant disruption of endogenous biochemical pathways, which can enhance spatiotemporal control and minimize unwanted toxicity [15,73,74].

Advanced catalytic nanoparticles are designed to incorporate targeting ligands, biomimetic coatings, biodegradable polymers, and stimuli-responsive structures to enhance the targeting of the disease site and the therapeutic specificity of the nanoparticles. Multifunctional nanozyme platforms can be used for multiple purposes, such as imaging, drug delivery, immune regulation, metabolic modulation, and catalysis. Renal targeting ligands, cell membrane coating, and size/charge-optimized nanostructures could enhance renal targeting of glomerular, tubular, urinary, or reproductive tissues. These design principles enable the catalytic activity to be matched to the pathological requirements of a given disease while avoiding off-target oxidative injury and systemic toxicity. Thus, catalytic nanotherapeutics are versatile platforms for precision urology, nephrology, and reproductive medicine [16,75,76].

### 2.4 Reactive oxygen modulation strategies

The key therapeutic mechanism of NCM in urological and reproductive diseases is the bidirectional modulation of ROS. Catalytic ROS amplification in malignant tissues leads to elevated oxidative stress, mitochondrial dysfunction, membrane damage, protein oxidation, DNA fragmentation, and apoptotic or ferroptotic death. Biocatalytic nanomaterials can catalyze the production of hydroxyl radicals, singlet oxygen, and superoxide radicals through reactions with endogenous and/or exogenous substrates. These pro-oxidant therapies can enhance the effectiveness of chemotherapy, radiotherapy, phototherapy, and immunotherapy and may overcome resistance to these therapies in ovarian, prostate, bladder, renal, and other reproductive cancers [[77], [78], [79]].

Other tumor-specific approaches are based on the generation of oxygen and depletion of GSH, which alters redox homeostasis to boost oxidative therapy. CAT-mimicking nanozymes can alleviate hypoxia thereby increasing the efficiency of photodynamic and oxidative therapy. Glutathione depletion decreases the ability to remove ROS from cells, leading to increased cell damage by ROS. Inducing lipid peroxidation and ferroptosis in resistant malignant cells appears to be a novel and effective method for killing these cells [[80], [81], [82]]. In contrast, in situations such as nephritis, nephrotic syndrome, acute kidney injury, and non-cancerous conditions of reproduction, excessive production of ROS leads to cell damage, inflammation, vascular dysfunction, and fibrosis. Under these conditions, SOD-, CAT-, GPx-, and antioxidant-like nanozymes can neutralize reactive intermediates, maintain mitochondrial functionality, and decrease inflammation-induced tissue damage [19,32,83].

Stimuli-responsive and biomimetic nanozyme systems allow control of the dynamics of ROS in response to the pathological conditions of the environment. With these platforms, pro-oxidant catalysis may be induced in malignant tissues, and antioxidant and anti-inflammatory effects may be induced in damaged renal and reproductive tissues. Multifunctional ROS-regulating nanomaterials can also be combined with immunotherapy, phototherapy, metabolic therapy, anti-inflammatory therapy, regenerative therapy, and imaging techniques for monitoring. Thus, accurate, two-way, and disease-specific ROS regulation is a fundamental approach for the treatment of urological and reproductive diseases using nanocatalysts [[84], [85], [86]].

## 3 Biocatalytic nanomaterials for urological, kidney, and reproductive diseases

### 3.1 Metal-based biocatalytic nanomaterials

**Fig. 2:**
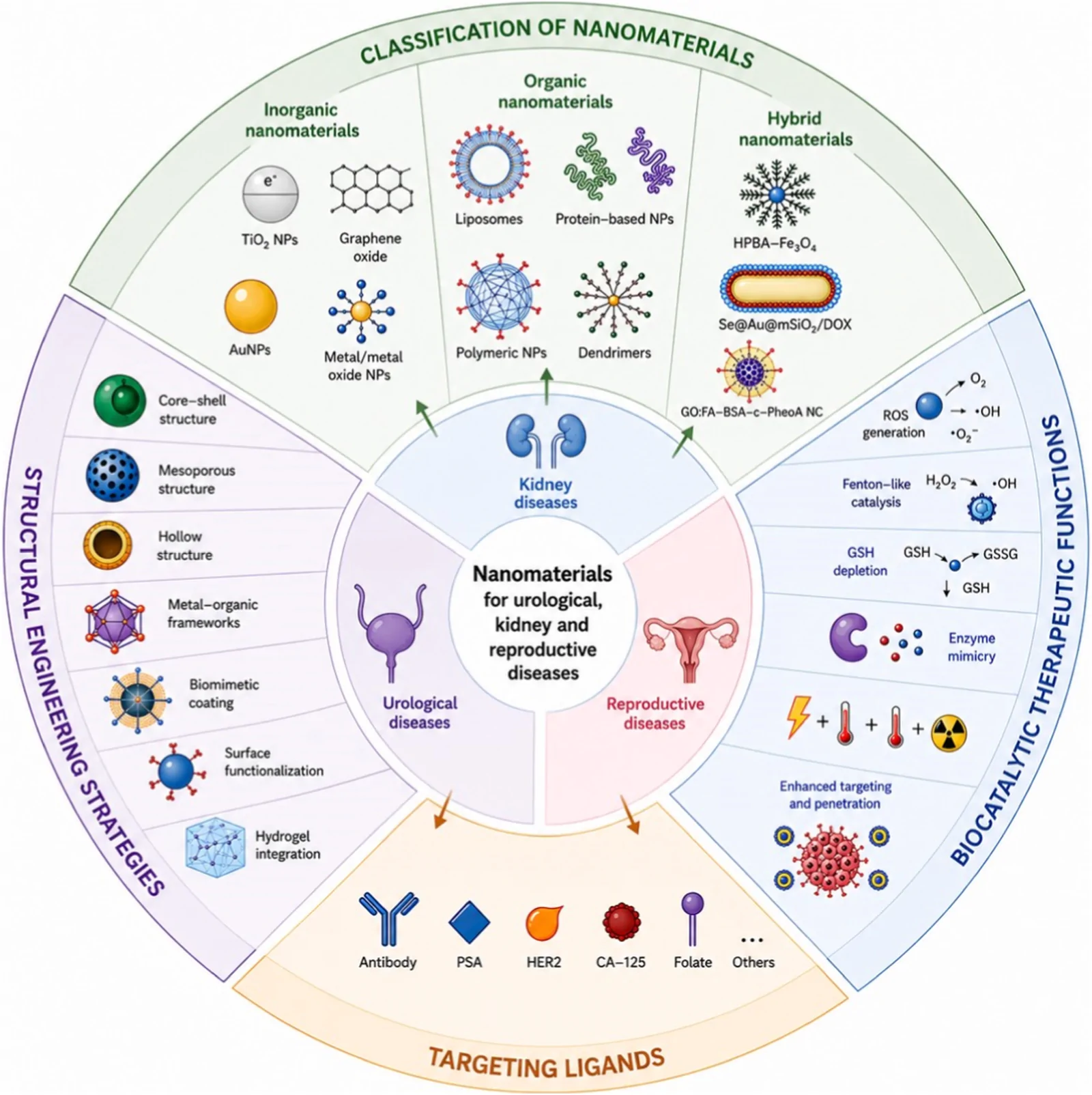
Classification, structural engineering strategies, targeting ligands, and biocatalytic therapeutic functions of inorganic, organic, and hybrid nanomaterials in urological, kidney, and reproductive diseases. The image was created using BioRender.

Metal-based biocatalytic nanomaterials exhibit high catalytic efficiency, redox responsiveness, and multifunctional therapeutic potential, making them promising platforms for the treatment of urological, kidney, and reproductive diseases. The biochemical reactions involved in pathologies can be regulated by transition metal nanozymes, which are involved in ROS generation and scavenging. Their catalytic activity can be tuned by oxidation state control, reaction kinetic optimization, and surface area engineering. Major inorganic nanoparticles include iron (Fe), copper (Cu), manganese (Mn), cobalt (Co), platinum (Pt), gold (Au), and titanium (Ti)-based nanoparticles, and their related nanozymes. The structural engineering and therapeutic mechanisms of these compounds are summarized in Fig. 2 [[87], [88], [89]].

In urologic and reproductive cancers, such as renal, bladder, prostate, ovarian, cervical, and endometrial cancers, iron-based nanozymes can promote Fenton or Fenton-like reactions to convert endogenous H_2_O_2_ into cytotoxic hydroxyl radicals. Under the acidic conditions of a tumor, copper-containing systems also experience redox cycling of Cu^+^/Cu^2+^, increase ROS production, reduce GSH levels, and impair mitochondrial function. Nanomaterials with oxidase, peroxidase, CAT, and photothermal activities include manganese, cobalt, platinum, and gold-based materials, which can control hypoxia, improve imaging, and provide combination therapy [21,35,72].

Metal-based nanozymes can be engineered to remove superoxide, H_2_O_2_, and other reactive intermediates to reduce oxidative stress in kidney diseases, such as nephritis, nephrotic syndrome, acute kidney injury, chronic kidney disease, and renal ischemia-reperfusion injury. Nanozymes based on cerium, manganese, and platinum can have SOD-, CAT-, or GPx-like activity to minimize oxidative damage to the tubules and glomeruli, podocyte damage, mitochondrial dysfunction, inflammation, and renal fibrosis. To maximize the accumulation of nanocarriers in the kidneys, their surfaces can be functionalized with surface ligands, biomimetic coatings, and renal targeting ligands, which can further enhance their catalytic stability, circulation time, and biosafety. Thus, metal-based nanozymes can be used for versatile applications, such as the catalytic degradation of pathological cells and the protection of damaged kidneys and reproductive tissues [19,40,90].

### 3.2 Metal oxide and hybrid catalytic nanostructures

The tunable catalytic activity, structural stability, and multifunctional biological performance of metal oxide nanozymes and hybrid catalytic nanostructures render them highly versatile therapeutic platforms. These catalytic nanomaterials can effectively control ROS generation, oxygen production, and glutathione reduction, maintaining intracellular redox homeostasis. Their porous architectures and tunable surfaces enable high drug loading, disease-responsive release, targeted delivery, and integration with imaging or externally activated therapy [91,92].

**Fig. 3:**
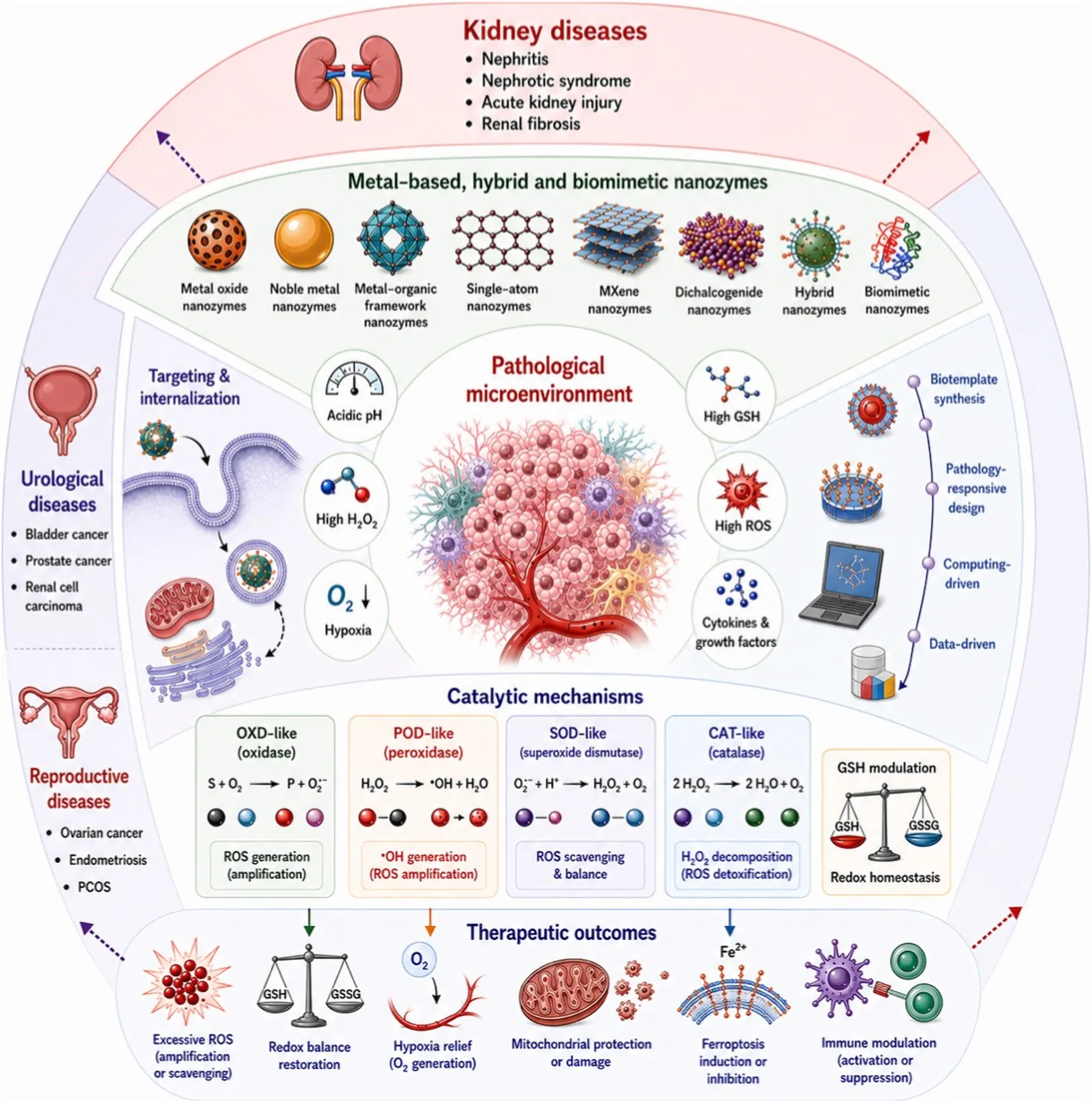
Catalytic mechanisms of metal-based, hybrid, and biomimetic nanozymes in the pathological microenvironments of urological, kidney, and reproductive diseases highlight the oxidase, peroxidase, SOD, and CAT-like activities involved in ROS amplification or scavenging, GSH modulation, hypoxia regulation, ferroptosis induction or inhibition, mitochondrial damage or protection, and immune modulation. Image was created with BioRender. The concept was adapted from previous studies [95,96].

The peroxidase-like activity of Fe_3_O_4_ nanoparticles makes them a potential tool for chemodynamic and ferroptosis therapies for renal, urinary, and reproductive cancers. MnO_2_ nanozymes can decompose H_2_O_2_ to generate oxygen, relieve hypoxia, and consume glutathione. In contrast, redox-active metal oxides, such as CeO_2_, can be involved in reversible valence cycling, remove excess ROS, and are relevant in nephritis, nephrotic syndrome, acute kidney injury, chronic kidney disease, and inflammatory reproductive disorders. Depending on the nature of the disease, a choice may be made among the following activities: oxidase, peroxidase, SOD, CAT, and GPx-like activities. These catalytic pathways control mitochondrial damage, ferroptosis, inflammation, immune activation, fibrosis, and repair (Fig. 3) [93,94].

Hybrid catalytic nanostructures are composed of metal oxides, polymers, biomolecules, liposomes, carbon-based materials, and therapeutic agents to enhance targeting, catalytic control, and biological compatibility. These hybrid systems often feature ROS generation, photothermal or photodynamic effects, controlled drug delivery, and biomarker targeting. In the case of kidney and nonmalignant reproductive diseases, hybrid systems could also contain antioxidant nanozymes, anti-inflammatory or antifibrotic agents, renal-targeting ligands, and biodegradable carriers to inhibit oxidative damage and maintain organ functions. Therefore, hybrid catalytic nanostructures can be used in precision therapy for specific diseases in urology, nephrology, and reproductive medicine [61,97].

### 3.3 Carbon-based and polymeric biocatalytic nanomaterials

Carbon-based and polymeric biomaterials have received considerable attention owing to their flexible structures, tunable biocompatibility, and versatility in surface functionalization. Graphene oxide, carbon nanotubes, carbon dots, and conductive carbon nanostructures have optical, electronic, catalytic, and photothermal properties that can be utilized to control the generation of ROS, drug delivery, and metabolic pathways in cells. For renal, urinary, and reproductive cancers, these materials can be used to deliver therapeutic cargo intracellularly, induce photothermal conversion, catalyze ROS amplification, and integrate multimodal therapy. For kidney injury, nephritis, nephrotic syndrome, and inflammatory reproductive disorders, carbon nanomaterials can be designed to capture ROS, maintain mitochondrial function, and provide anti-inflammatory, antioxidant, and regenerative molecules [98,99].

Polymeric catalytic nanoplatforms offer tunable biodegradability, controlled therapeutic release, and increased physiological stability. Polymeric nanoparticles, dendrimers, protein-based carriers, hydrogels, and liposomal systems can be designed to respond to the acidic environment, hypoxia, high ROS levels, enzymes, or inflammatory mediators encountered in diseased tissues. In tumors, ROS-responsive polymers can disrupt the intracellular redox balance and increase oxidative damage in tumor tissues. These polymers can scavenge reactive species or release antioxidants, anti-inflammatory, immunomodulatory, and antifibrotic agents [53,100,101].

Carbon-based and polymeric platforms can be conjugated with peptides, antibodies, aptamers, and receptor-targeting ligands to increase kidney, urinary tract, and reproductive tissue localization. Potential improvements in the accumulation in glomeruli, podocytes, proximal tubules, or even inflamed kidney tissue are possible with peptide and charge- or size-optimized carriers. Multifunctional systems that combine catalysis, drug/gene delivery, imaging, phototherapy, immune regulation, and renal protection hold great promise for the precision treatment of urological, renal, and reproductive diseases [98,102,103].

### 3.4 Biomimetic and bioengineered catalytic nanomaterials

Biomimetic and bioengineered catalytic nanomaterials are designed with consideration of natural biological structures, tissue-targeting interfaces, and enzymatic functions to enhance the tissue targeting, catalytic activity, physiological stability, and therapeutic compatibility of catalytic nanomaterials. Compared with conventional nanomedicines, these systems can evade rapid immune clearance, prolong systemic circulation, enhance cellular uptake, and facilitate selective interactions with pathological tissues in urological, renal, and reproductive diseases. Target tissue-specific cell membrane coatings, extracellular vehicles (EVs), protein-based carriers, and biologically derived templates can be selected depending on the tissue (such as tumors, glomeruli, renal tubules, urinary tissue, and reproductive organs) [104,105].

Natural membrane proteins that have immune-evasive properties, as well as other functions such as homotypic targeting, intercellular communication, and intercellular tissue targeting, are incorporated into nanozymes and exosome-like catalytic systems through the cell membrane. Tumor or immune cell membrane coatings can further promote accumulation in cancerous tissues and facilitate ROS amplification, ferroptosis, delivery, and immune activation in renal, urinary, and reproductive cancers. The membranes of erythrocytes, platelets, macrophages, and stem cells can be useful for renal localization, decreasing inflammatory recognition, and allowing the delivery of antioxidant or anti-inflammatory nanozymes in nephritis, nephrotic syndrome, acute kidney injury, and renal ischemia–reperfusion injury. Incorporating natural enzymatic activity with synthetic catalytic activity can increase the level of ROS in tumors or decrease excessive ROS in damaged kidneys and reproductive tissues using enzyme-loaded nanomaterials [[106], [107], [108]].

Active site biomimetization, biomimetic active site engineering, biomimetic synthesis via biotemplated assembly, cascade catalysis, and pathology-responsive nanozyme design further enhance catalytic selectivity and therapeutic adaptability (Fig. 3). All of these tools - imaging, drug delivery, immune regulation, metabolic modulation, and catalytic therapy - can be integrated into a single system on a bioengineered platform. Biomolecular ligands, such as peptides, antibodies, and aptamers, can be used for surface functionalization to enhance the recognition of tumor biomarkers, renal tubular receptors, podocytes, inflammatory cells, and reproductive tissue targets. Thus, biology-inspired catalytic nanomaterials have great potential in precision oncology, renal protection, inflammation regulation, and tissue regeneration in urological, renal, and reproductive disorders [[109], [110], [111]].

### 3.5 Stimuli-responsive catalytic nanomaterials

The development of stimuli-responsive catalytic nanomaterials has made it possible to selectively activate therapeutic functions in response to the biochemical and physical properties of urological, kidney, and reproductive diseases. These systems can control ROS generation or removal, catalytic ion release, oxygen generation, and therapeutic cargo delivery in response to acidic pH, hypoxia, increased H_2_O_2_ concentration, changes in GSH levels, inflammatory enzymes, oxidative stress, light, ultrasound, magnetic fields, and temperature changes. This responsiveness improves disease site specificity and minimizes unwanted catalytic activity and toxicity in healthy tissues [18,112].

For urological and reproductive cancers, pH- and H_2_O_2_-responsive nanoplatforms exploit the acidic and oxidatively dysregulated tumor microenvironments (TMEs) and induce Fenton or Fenton-like reactions to enhance ROS production. Hypoxia-responsive nanozymes can produce oxygen and enhance PDT, CDT, and immunocatalytic therapy (ICT). Ultrasound- or magnetically responsive nanozymes can catalyze ROS generation while enabling externally controlled and spatially localized therapeutic activation of the tumor. Light-activated systems can be either catalytic ROS-generating systems or photothermal therapy (PTT) systems [[113], [114], [115]].

Stimuli-responsive systems could be activated to target antioxidant and anti-inflammatory responses only where there is an excess of ROS, low pH, hypoxia, and/or high enzyme activity, such as in kidney diseases like nephritis, nephrotic syndrome, acute kidney injury, chronic kidney disease and ischemia-reperfusion injury. ROS-responsive polymers can scavenge ROS and release antioxidant, anti-inflammatory, or antifibrotic agents. Enzyme-responsive carriers can modulate inflammatory or proteolytic pathways, whereas hypoxia-responsive carriers can protect renal tubular and endothelial cells by ensuring proper oxygen balance and mitochondrial function [101,116,117].

The multifunctionality and adaptability of catalytic therapy are boosted by the multiple responsive mechanisms of a single nanoplatform. Smart systems can be combined with ROS amplification/scavenging, controlled drug release, mitochondrial regulation, induction or inhibition of ferroptosis, immune modulation, and imaging guidance. Responsive biomimetic coatings and intelligent polymeric structures can be used to further enhance the circulation stability, accumulation of nanocarriers in the kidneys or tumors, and site-specific activation of nanocarriers. Hence, stimuli-responsive catalytic nanomaterials can be proposed as promising platforms for the personalized treatment of urological, kidney, and reproductive diseases [53,112,118].

## 4 Nanocatalytic therapeutic strategies in urological, kidney, and reproductive diseases

### 4.1 Disease-specific applications across GU malignancies

Although ovarian cancer has served as the principal model for the development of NCM, increasing evidence demonstrates that similar catalytic strategies are applicable across a broad spectrum of GU malignancies, including bladder, renal cell, prostate, cervical, endometrial, and upper tract urothelial carcinomas. Despite differences in tissue origin and molecular characteristics, these malignancies share common redox-associated vulnerabilities, including oxidative stress, elevated H_2_O_2_ levels, GSH-dependent antioxidant defenses, hypoxia, metabolic reprogramming, and immunosuppressive tumor microenvironments. Consequently, catalytic therapeutic approaches, including CDT, catalytic phototherapy, ferroptosis induction, metabolic modulation, immunocatalytic therapy, and theranostic platforms, can be adapted to exploit these shared biochemical features while incorporating disease-specific targeting strategies for effective treatments. Therefore, the following sections discuss these therapeutic modalities from a mechanism-based perspective, highlighting representative applications across diverse GU malignancies.

### 4.2 Chemodynamic therapy

**Fig. 4:**
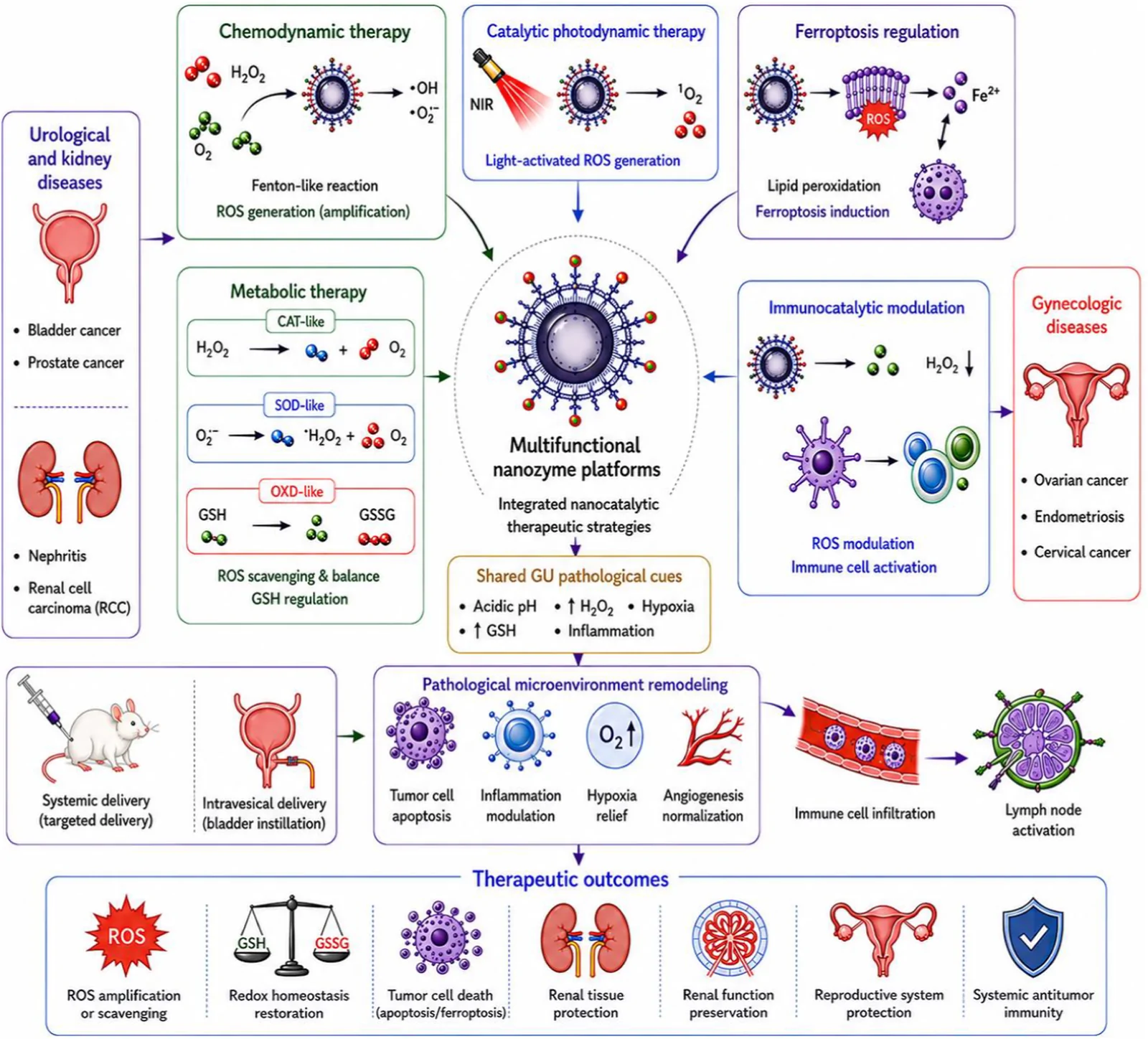
Multifunctional nanozyme platforms for the integrated treatment of urological, kidney, and gynecologic diseases. Nanozymes combine CDT, PDT, metabolic, ferroptotic, and immunocatalytic mechanisms to remodel pathological microenvironments, regulate redox balance, promote tumor cell death, protect renal and reproductive tissues, and enhance systemic antitumor immunity. Image created with BioRender; concept adapted from previous studies [123,124].

CDT involves the use of Fenton or Fenton-like reactions to generate cytotoxic ROS in pathological tissues, notably in renal, urinary, and reproductive cancers. Transition metal-based nanozymes can convert endogenous hydrogen peroxide to highly reactive hydroxyl radicals and superoxide species, thereby enhancing oxidative stress and tumor-specific cytotoxicity [119,120]. Acidic pH and elevated H_2_O_2_ levels can facilitate Fenton/Fenton-like catalysis, whereas high GSH levels and hypoxia may restrict treatment efficacy, requiring concurrent GSH depletion or oxygen-regulating strategies. As shown in Fig. 4, CDT-associated nanoplatforms increase ROS generation and destroy cancer cells [121,122]**.**

Although ovarian cancer remains the most extensively investigated model for nanocatalytic CDT, comparable therapeutic strategies have been reported for bladder cancer, RCC, prostate cancer, cervical cancer, endometrial cancer, and upper tract urothelial carcinoma, reflecting the shared dependence of these malignancies on acidic, H_2_-rich, and GSH-regulated tumor microenvironments.

Overproduction of intracellular ROS during CDT can lead to lipid peroxidation in membranes, protein oxidation, and DNA fragmentation, which subsequently induce apoptosis, ferroptosis, and necrosis. Restricting ROS production to tumorous tissues could increase therapy selectivity, provided that the production is tumor-specific. Catalytic nanoplatforms exhibit oxidase-, CAT-, and SOD-like activities, further improving their therapeutic performance under oxidative conditions [125,126].

Biomimetic coatings, tissue-targeting ligands, and stimuli-responsive polymers have significantly enhanced the catalytic stability, circulation, and accumulation of drugs in urological and reproductive tumors in recent years. These multifunctional systems that integrate ROS enhancement, hypoxia control, chemotherapy, ferroptosis, imaging, and immune activation could further enhance the treatment of resistant malignancies. In nephritis, nephrotic syndrome, acute kidney injury, and other nonmalignant inflammatory diseases, where ROS accumulation may exacerbate renal injury, pro-oxidant CDT should be used cautiously (Table 2). Therefore, it is crucial to have catalytic control of disease-specific therapy to ensure safety in precision therapy [101,117]**.**

**Table 2:** Representative biocatalytic nanomaterials, therapeutic modalities, targeting strategies, and translational outcomes in urological, kidney, and reproductive diseases.

Representative nanocatalytic system	Therapeutic modality	Targeting/responsive strategy	Major therapeutic function	Reported preclinical outcome	Ref
Fe_3_O_4_ nanozymes	CDT	Acidic and H_2_O_2_-rich microenvironment responsiveness	Hydroxyl-radical generation and ferroptosis induction	Enhanced destruction of renal, urinary, and reproductive tumors	[21,127]
Cu-based catalytic nanoparticles	Fenton-like catalytic therapy	Redox-responsive Cu^+^/Cu^2+^ cycling	ROS amplification and GSH depletion	Improved cytotoxicity	[35,128]
MnO_2_ nanozymes	PDT and oxygen-regulating therapy	Hypoxia- and H_2_O_2_-responsive activation	Oxygen generation, hypoxia alleviation, and ROS regulation	Improved phototherapy and tissue oxygenation	[35,129]
Au-based photothermal nanozymes	Photothermal–catalytic therapy	Near-infrared responsiveness and ligand-mediated targeting	Combined thermal injury and ROS amplification	Enhanced ablation of localized urological and reproductive tumors	[130,131]
CeO_2_ catalytic nanoplatforms	Redox modulation	ROS-responsive Ce^3+^/Ce^4+^ switching	Scavenging of superoxide and H_2_O_2_	Reduced oxidative injury, inflammation, and fibrosis in kidney and reproductive tissues	[132,133]
Pt-based antioxidant nanozymes	Renoprotective catalytic therapy	Oxidative-stress-responsive enzyme mimicry	SOD- and CAT-like ROS scavenging	Protection against acute kidney injury and ischemia–reperfusion damage	[134,135]
GOx-loaded nanozymes	Starvation therapy	Metabolic and glucose-responsive activation	Glucose depletion, H_2_O_2_ generation, and ATP reduction	Metabolic collapse of malignant cells	[47,136]
Ferroptosis-inducing iron nanozymes	Ferroptotic catalytic therapy	GSH depletion and GPx4 suppression	Lipid-peroxidation amplification	Elimination of resistant tumors	[58,137]
Ferroptosis-inhibiting antioxidant nanozymes	Renal and reproductive tissue protection	ROS- and lipid-peroxide-responsive activation	Suppression of lipid peroxidation and mitochondrial injury	Reduced tubular, glomerular, and reproductive tissue damage	[138,139]
Renal-targeted antioxidant nanozymes	Kidney-protective nanocatalytic therapy	Size-, charge-, peptide-, or receptor-mediated renal targeting	ROS scavenging and inflammatory suppression	Improved treatment of nephritis, nephrotic syndrome, and kidney injury	[19]
Biomimetic membrane-coated nanozymes	Precision catalytic therapy	Cell-membrane-mediated targeting	Disease-site accumulation and immune-evasive delivery	Reduced off-target toxicity and improved therapeutic selectivity	[140,141]
Exosome-inspired catalytic systems	Biohybrid catalytic delivery	Natural vesicle-mediated transport	Intracellular redox regulation and therapeutic delivery	Improved penetration and biocompatibility in renal and reproductive tissues	[142,143]
pH-responsive polymeric nanozymes	Smart catalytic therapy	Acidic-pH-triggered activation	Controlled ROS generation, scavenging, or drug release	Pathology-selective therapeutic activation	[144,145]
Hypoxia-responsive catalytic nanoplatforms	Oxygen-regulated therapy	Activation in oxygen-deficient tissues	Oxygen generation and redox modulation	Improved tumor phototherapy and protection of ischemic tissues	[56,146]
Immunocatalytic nanozymes	Catalytic immunotherapy or anti-inflammatory therapy	Immune-microenvironment modulation	ICD or suppression of pathological inflammation	Enhanced antitumor immunity or reduced renal and reproductive inflammation	[147,148]
Theranostic catalytic nanoplatforms	Imaging-guided catalytic therapy	MRI, ultrasound, or photoacoustic responsiveness	Integrated diagnosis, catalytic treatment, and response assessment	Real-time treatment monitoring	[149,150]
Multifunctional hybrid nanozymes	Multimodal catalytic therapeutics	Stimuli-responsive multimodal activation	Combined ROS regulation, drug delivery, immune modulation, and imaging	Improved precision treatment across urology, nephrology, and reproductive medicine	[9,151]

### 4.3 Catalytic photodynamic and photothermal therapies

Catalytic PDT and PTT combine light-activated therapeutic action with catalytic control of ROS and oxygen levels. Photosensitizer-loaded catalytic nanoplatforms produce singlet oxygen and other reactive species upon light irradiation, allowing for the light-induced destruction of renal, bladder, prostate, ovarian, cervical, and other reproductive tumors. An important limitation of conventional PDT is tumor hypoxia, which can be overcome by using CAT-like nanozymes that decompose H_2_O_2_ to produce oxygen and reduce tumor hypoxia [152,153]**.**

Despite differences in tissue origin, these malignancies exhibit similar hypoxic and oxidative tumor microenvironments, allowing catalytic phototherapies to exploit common ROS-dependent therapeutic mechanisms while requiring disease-specific optimization of the targeting strategies.

Duan et al. and Nasseri et al. showed that photothermal catalytic therapy have increased the local temperatures that can enhance the catalytic reaction rate, increase membrane permeability and cellular uptake, and enhance the disruption of tumor metabolism by ROS. Photothermal platforms also enable spatiotemporal monitoring and delivery of treatment, which can be achieved using imaging-guided and stimulus-responsive platforms [154,155].

Therefore, a hybrid nanoplatform with PDT, PTT, and CDT can generate heat, increase oxidative stress, cause mitochondrial damage, trigger ferroptosis, and change the immune microenvironment [156]. For nonmalignant kidney and reproductive diseases, the systems could be designed to release controlled amounts of a drug, have antimicrobial properties, or deliver a localized amount of antioxidants; however, in this case, the parameters of the externally applied irradiation would need to be optimized to avoid thermal and oxidative damage to sensitive tissues. Hence, catalytic phototherapy is a promising externally controlled method for precision therapy, especially for localized tumors of the urological and reproductive systems [[157], [158], [159]].

### 4.4 Ferroptosis-regulating nanocatalytic systems

Owing to their involvement in various malignant and nonmalignant urological, kidney, and reproductive diseases, ferroptosis-regulating nanocatalytic systems have attracted considerable research interest. Ferroptosis induction eliminates susceptible cancer cells, particularly treatment-resistant cells, by promoting iron-dependent lipid peroxidation and membrane damage. The generation of hydroxyl radicals by iron-based nanozymes via Fenton chemistry leads to lipid peroxidation, irreversible oxidative damage, and tumor cell death [21,66,160].

Tumor response to ferroptosis can be enhanced by targeting iron metabolism and GSH-dependent antioxidant pathways. The loss of GSH and a decrease in GPx4 lead to a decrease in the protection of the cell against lipid peroxidation, while the presence of oxidase- and peroxidase-like activities results in an increased production of ROS. These mechanisms are particularly important in chemoresistant tumors, which exhibit elevated GSH levels and adaptability to metabolic changes [[161], [162], [163]].

Ferroptosis-inducing nanozymes have shown promise in ovarian, bladder, prostate, and RCC, where elevated GSH-dependent antioxidant defenses, metabolic plasticity, and therapeutic resistance create favorable conditions for catalytic lipid peroxidation. These observations support the broader applicability of ferroptosis-based nanocatalytic strategies in GU malignancies, despite their distinct tissue origins.

However, excessive ferroptosis leads to renal tubular damage, ischemia–reperfusion damage, nephrotoxicity, inflammatory kidney damage, and reproductive tissue disorders. In such situations, antioxidant nanozymes can suppress lipid peroxidation, maintain normal GSH metabolism, protect mitochondria, and inhibit ferroptosis. Therefore, catalytic nanoplatforms must be engineered to either trigger ferroptosis in cancerous tissue or inhibit it in sensitive cells of the kidney and reproductive organs. Surface-modified and biomimetic systems can enhance tissue specificity and minimize unwanted damage [161,164,165].

### 4.5 Catalytic metabolic regulation and starvation therapy

Catalytic metabolic modulation has been investigated in ovarian, bladder, prostate, renal cell, cervical, and endometrial cancers because these malignancies share increased metabolic demands, mitochondrial dysfunction, and redox-associated metabolic rewiring. Catalytic metabolic modulation has been investigated in ovarian, bladder, prostate, renal cell, cervical, and endometrial cancers because these malignancies share increased metabolic demands, mitochondrial dysfunction, and redox-associated metabolic rewiring.

The therapeutic approach of catalytic starvation therapy focuses on the metabolism of nutrients and energy and is mainly used in urological and reproductive cancers. Glucose oxidase-loaded nanoplatforms remove glucose and oxygen from tumors and generate gluconic acid and H_2_O_2_ [166]. This process creates nutrient deprivation, decreases ATP production, increases acidosis, and provides a source of H_2_O_2_ for subsequent Fenton or Fenton-like reactions to generate ^•^OH. Combined metabolic and oxidative stress inhibits tumor cell proliferation, migration, and survival [47,167].

In addition, catalytic nanomedicine can control mitochondrial respiration, glycolysis, glutamine utilization, lipid metabolism, and redox-related biosynthetic pathways. Nanozymes with CAT activity can regulate the availability of H_2_O_2_ and oxygen, those with SOD activity can control the conversion of superoxide to H_2_O_2_ and oxidase-like materials can be used to increase the depletion of substrates or the oxidation of GSH. These catalytic reactions can lead to metabolic failure in cancer [[168], [169], [170]].

For nonmalignant kidney and reproductive diseases, metabolic interventions should be geared towards restoring, not depleting, cellular energy. Nanozymes can preserve mitochondrial respiration, decrease oxidative metabolic stress, enhance oxygen use, and prevent fibrosis and tissue degeneration. Disease-appropriate treatments can be provided by responsive systems that regulate metabolism and control ferroptosis, modulate the immune response, and enable imaging-based monitoring. The use of catalytic starvation is still suitable for tumors, whereas metabolic normalization is suitable for nephritis, nephrotic syndrome, kidney injury, and degenerative reproductive diseases [161,171,172].

### 4.6 Immunocatalytic nanomedicine

Immunocatalytic nanomedicine combines catalytic redox regulation with the modulation of innate and adaptive immunity. Catalytic nanozymes generate ROS, leading to immunogenic cell death, the release of tumor-associated antigens, and damage-associated molecular patterns (DAMPs). These immunostimulatory signals guide the maturation of dendritic cells, activation of lymph nodes, presentation of antigens, and activation of cytotoxic T cells. The connection between the catalytic destruction of tumors, immune cell infiltration, and systemic antitumor immunity is shown in Fig. 4 [173,174].

TAMs can also be reprogrammed from an immunosuppressive to a proinflammatory antitumor state using catalytic nanozymes. Oxygen-generating and GSH-depleting systems can be used to reduce hypoxia-associated immune suppression and facilitate immune cell infiltration. Combination therapy with immune checkpoint blockade has the potential to further augment antigen-specific responses, long-term tumor control, and immune memory [175,176].

ROS-scavenging immunocatalytic systems have been investigated preclinically for acute kidney injury, chronic kidney disease, and inflammatory reproductive disorders, but involve a therapeutic approach in the opposite direction. By inhibiting the excessive activation of macrophages and neutrophils, ROS-scavenging nanozymes can limit the production of inflammatory cytokines, safeguard renal and reproductive cells, and reduce fibrosis. Tumor immunotherapy or anti-inflammatory treatment of disease may then be tailored to the context of the disease by designing biomimetic and stimuli-responsive systems to activate the antitumor immune response or dampen pathological inflammation. This immune regulation is bidirectional and provides a wide range of new applications for immunocatalytic medicine beyond oncology [134,177].

### 4.7 Combination and multimodal catalytic therapeutics

New combination and multimodal catalytic therapy approaches have been designed to overcome the biological heterogeneity, clinical resistance, and complex pathological microenvironments of urological, kidney, and reproductive diseases. In cancer, catalytic nanoplatforms can also be used to increase the intracellular accumulation of drugs when used in combination with chemotherapy, improve oxidative sensitivity, and decrease multidrug resistance. Synergistic catalytic activation of drugs can enhance the efficiency of tumor destruction, allow the administration of drugs at lower concentrations, and decrease systemic toxicity [178,179].

Catalysis is further coupled with immunotherapy, PDT, PTT, ferroptosis regulation, metabolic therapy, gene delivery, and anti-inflammatory treatment, which can further expand its therapeutic functionality. Multifunctional nanozymes simultaneously enhance ROS production, modulate oncogenic signaling, deliver therapeutic genes, trigger ferroptosis, and promote antitumor immunity. Similar combinations may be applied to kidney and non-malignant reproductive diseases by integrating ROS scavenging, mitochondrial protection, anti-inflammatory and anti-fibrotic therapy, and tissue regeneration [9,180].

Theranostic catalytic nanoparticles integrate molecular imaging, tissue targeting, treatment activation, and real-time monitoring into a single platform. Smart nanozymes can be designed to be pro-oxidative, destroying malignant tissues; however, they can also be antioxidative and protective in inflamed and injured tissues. CDT, phototherapy, induction/inhibition of ferroptosis, metabolic regulation, immune modulation, and imaging-guided therapies can be included in disease-responsive platforms. Thus, multimodal catalytic therapeutics are a promising basis for personalized therapy in urology, nephrology, and reproductive medicine [181,182].

## 5 Precision therapeutics and smart nanocatalytic systems

### 5.1 Precision nanomedicine approaches

**Fig. 5:**
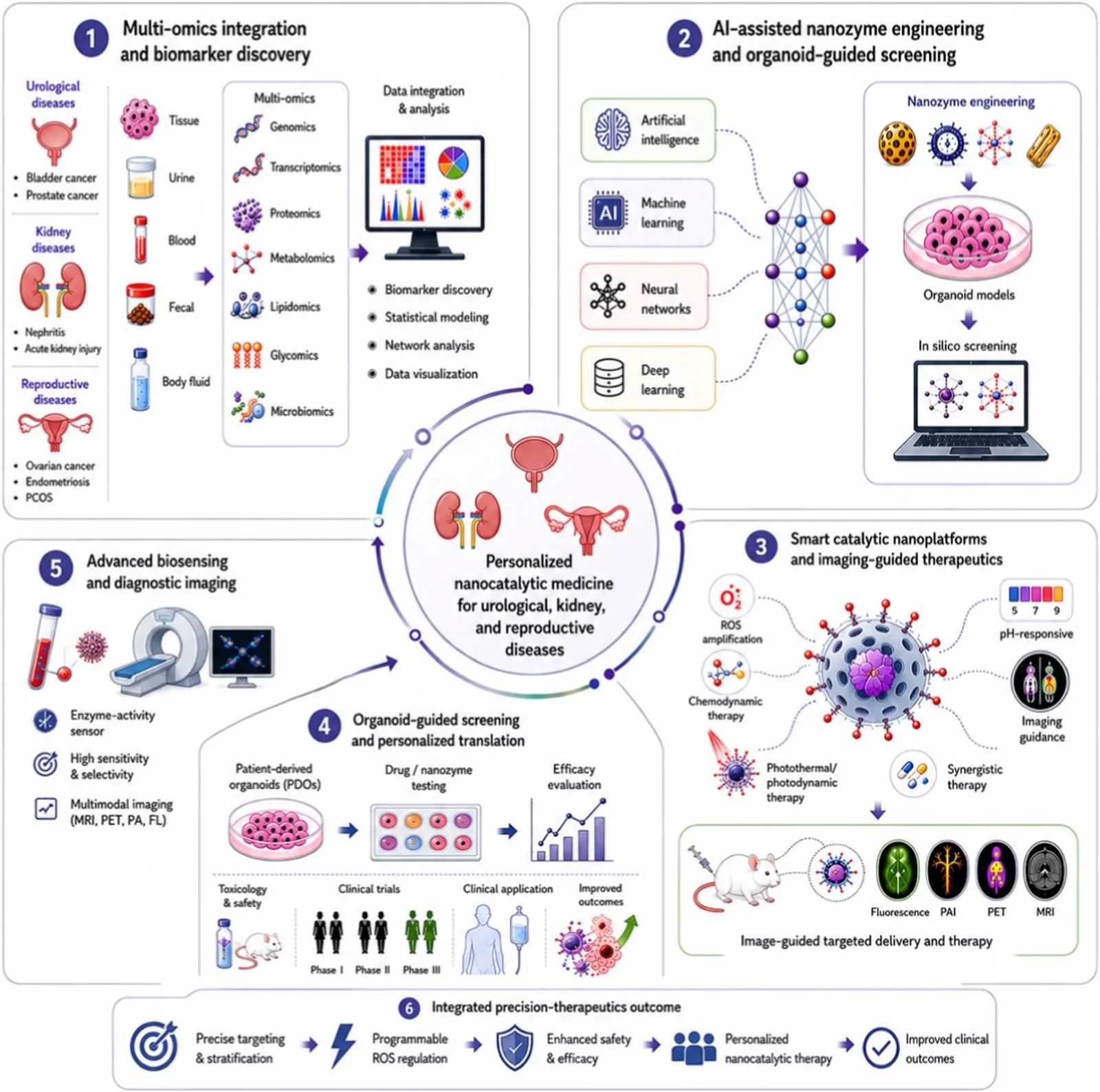
Precision NCM framework for urological, kidney, and reproductive diseases. Multi-omics biomarker discovery, AI-assisted nanozyme engineering, organoid-guided screening, smart imaging-enabled nanoplatforms, and advanced biosensing converge to enable patient stratification, programmable ROS regulation, improved safety, and personalized therapeutic outcomes. Image created with BioRender and concept adapted from previous studies [185,186].

Precision nanomedicine can be used to create customized catalytic systems specific to the molecular and pathological features of urological, kidney, and reproductive diseases. These diseases show significant differences in terms of genetic modifications, redox status, metabolic functions, tumor immune profile, tissue damage, fibrosis, and sensitivity to treatment [14]. Conventional therapies may not adequately address interpatient and disease-level heterogeneity, leading to suboptimal responses, recurrence of the condition, resistance, or worsening organ dysfunction. Catalytic nanomedicine provides disease site targeting, controlled regulation of ROS, metabolic modulation, biomarker-guided delivery systems, and local activation of therapeutic effects. A precision-therapeutics framework that combines multi-omics analysis, AI-guided nanozyme engineering, smart catalytic platforms, preclinical trials, and clinical translation is shown in Fig. 5 [183,184].

The use of biomarkers is crucial for enhancing the efficacy of treatments. Surface-engineered nanozymes with antibodies, peptides, aptamers, or receptor-targeting ligands can target and recognize the molecular markers of renal, urinary, and reproductive tissues. Tumor markers can be used for targeted ROS amplification in various cancers, including renal, bladder, prostate, ovarian, cervical, and endometrial cancers. Kidney injury markers, inflammatory mediators, and fibrosis-associated pathways can be used for targeted antioxidant or anti-inflammatory therapy in nephritis, nephrotic syndrome, acute kidney injury, and chronic kidney disease. The genetic, transcriptomic, proteomic, metabolomic, epigenetic, lipidomic, glycomic, and microbiomic features of diseases can define disease-specific vulnerabilities and redox pathways for the catalytic targeting of these diseases. Therapeutic selection and prediction of response to therapy are further supported by statistical modeling, pathway analysis, biomarker discovery, and network analysis, as reported by Castronovo et al. (2009) and Shanmugasundaram & Block (2016) [27,187].

Biospecimens, such as patient-derived tissues, blood, urine, and organoids, can be combined with computational platforms to create personalized catalytic therapy. Smart nanocatalytic systems can either enhance ROS levels in tumorous tissues or scavenge excess ROS in lesions associated with inflammation and ischemia. They may also react to changes in pH, hypoxia, enzymes, or metabolic abnormalities and have drug delivery, tissue protection, imaging, and immune modulation components. Hence, personalized nanomedicine holds great promise for the personalized treatment of urological, kidney, and reproductive diseases [145,188,189].

### 5.2 Pathology-responsive smart catalytic platforms

Smart catalytic platforms are designed to modulate therapeutic activities based on the biochemical and physiological properties of diseased tissues. Activation signals include acidic pH, hypoxia, increased H_2_O_2_ levels, changes in GSH levels, excess ROS, inflammatory enzymes, and metabolic dysfunction. These can be used to regulate the production or scavenging of ROS, release of catalytic ions, production of oxygen, drug delivery, and modulation of immune responses. This adaptive behavior enhances therapeutic precision and limits damage to healthy tissues compared to passive nanocarriers [[190], [191], [192]].

In urological and reproductive cancers, redox-responsive systems can decrease antioxidant defenses, increase oxidative stress, and induce apoptosis, ferroptosis, and ICD. Smart nanozymes can remove excessive ROS, downregulate inflammatory signaling, maintain mitochondrial function, and decrease fibrosis in kidney diseases and nonmalignant reproductive disorders. Catalytic adaptability can be further enhanced by multi-stimuli-responsive platforms, which incorporate a combination of pH-, enzyme-, ROS-, thermal-, hypoxia-, light-, or ultrasound-sensitive mechanisms that are disease-specific and activated only in the presence of disease conditions [97,193].

Intelligent release systems enable the spatiotemporal control of therapeutic agents, catalytic ions, antioxidants, antifibrotic drugs, and immunomodulatory molecules. The properties of responsive polymers and biomimetic coatings can be used to improve circulation stability, renal or tumor accumulation, cellular penetration, imaging guidance, and therapeutic efficacy. Multifunctional platforms capable of ROS regulation, photothermal/photodynamic activation, drug delivery, immune modulation, and imaging are important for the precise treatment of urological, kidney, and reproductive diseases [194,195].

### 5.3 AI-assisted and computational nanocatalytic design

AI-assisted nanocatalytic design offers an effective method for designing and optimizing NCMs for urological, kidney, and reproductive diseases. Machine learning algorithms can be used to analyze datasets describing the composition, catalytic activity, particle size, morphology, surface properties, biological interactions, biodistribution, toxicity, and therapeutic efficacy of nanomaterials. These methods can be used to determine the optimized configurations of nanozymes and optimize their catalytic activity, biological selectivity, stability, and biosafety [196].

Computational modeling can predict catalytic kinetics, ROS generation/scavenging dynamics, cell-cell interactions, drug release dynamics, and responses within the TME, renal, urinary, and reproductive microenvironments. The ability to simulate, model, analyze, and predict the molecular structures of nanozymes can be used to optimize their stability, substrate specificity, therapeutic responsiveness, and clearance. Machine learning, neural networks, and deep learning platforms can also aid in material screening, patient stratification, toxicity prediction, and the selection of disease-specific prooxidant or antioxidant catalytic functions [197,198].

The use of AI-assisted nanozyme engineering in conjunction with organoids, in silico screening, kidney-on-chip systems, patient-derived xenografts, and disease-specific animal models may increase preclinical reproducibility and clinical predictions. Fig. 5 shows the computational biology and digital medicine workflows that can be used in the design of nanozymes, therapeutic modeling, efficacy assessment, and personalized medicine. Consequently, the role of AI in precision urology, nephrology, and reproductive medicine is far-ranging [[199], [200], [201]].

### 5.4 Theranostic and imaging-guided catalytic nanomedicine

Theranostic and imaging-guided catalytic nanomedicine combines diagnostic imaging tools and therapeutic activation into a single multifunctional nanoplatform for the precision disease management. The ability of catalytic nanomaterials to image, target, and regulate ROS may prove beneficial for disease localization, monitoring, and personalized treatment planning for urological, kidney, and reproductive diseases. Fe-, Mn-, and Gd-based nanozymes can be used to improve magnetic resonance imaging contrast, and selected high-atomic-number or X-ray-attenuating formulations may additionally serve as CT contrast agents. Delivery to tumors, renal lesions, urinary tissues, and reproductive organs using imaging-guided platforms has the potential to be enhanced, and off-target exposure can be reduced [202].

In addition, the ability to track nanoplatform localization, catalytic activation, tissue oxygenation, and therapeutic responses can be enabled through the use of fluorescence, photoacoustic, ultrasound, radionuclide, and optical imaging, among others. Theranostic systems can visualize the targeting and amplification of ROS in cancer. In inflammatory reproductive pathologies and kidney injury, they can assess oxidative stress, renal perfusion, inflammation, fibrosis, and tissue recovery. These systems can be used to assist in the early evaluation of response, adjustment of doses, assessment of toxicity, and prediction of therapeutic outcomes [62,203].

Imaging-guided catalytic nanomedicine needs to be validated using patient-derived organoids, kidney and reproductive disease models, orthotopic tumor models, and physiologically relevant, *in vitro* platforms. However, comprehensive toxicity assays, pharmacokinetic evaluations, scalable manufacturing, and staged clinical trials remain essential. The future use of smart nanocatalytic systems, computational engineering, organoid screening, imaging-guided treatment, and precision therapeutic workflows will significantly improve the treatment of urological, kidney, and reproductive diseases [17,204].

## 6 Translational progress, biosafety, and clinical challenges

### 6.1 Preclinical evaluation of nanocatalytic systems

**Fig. 6:**
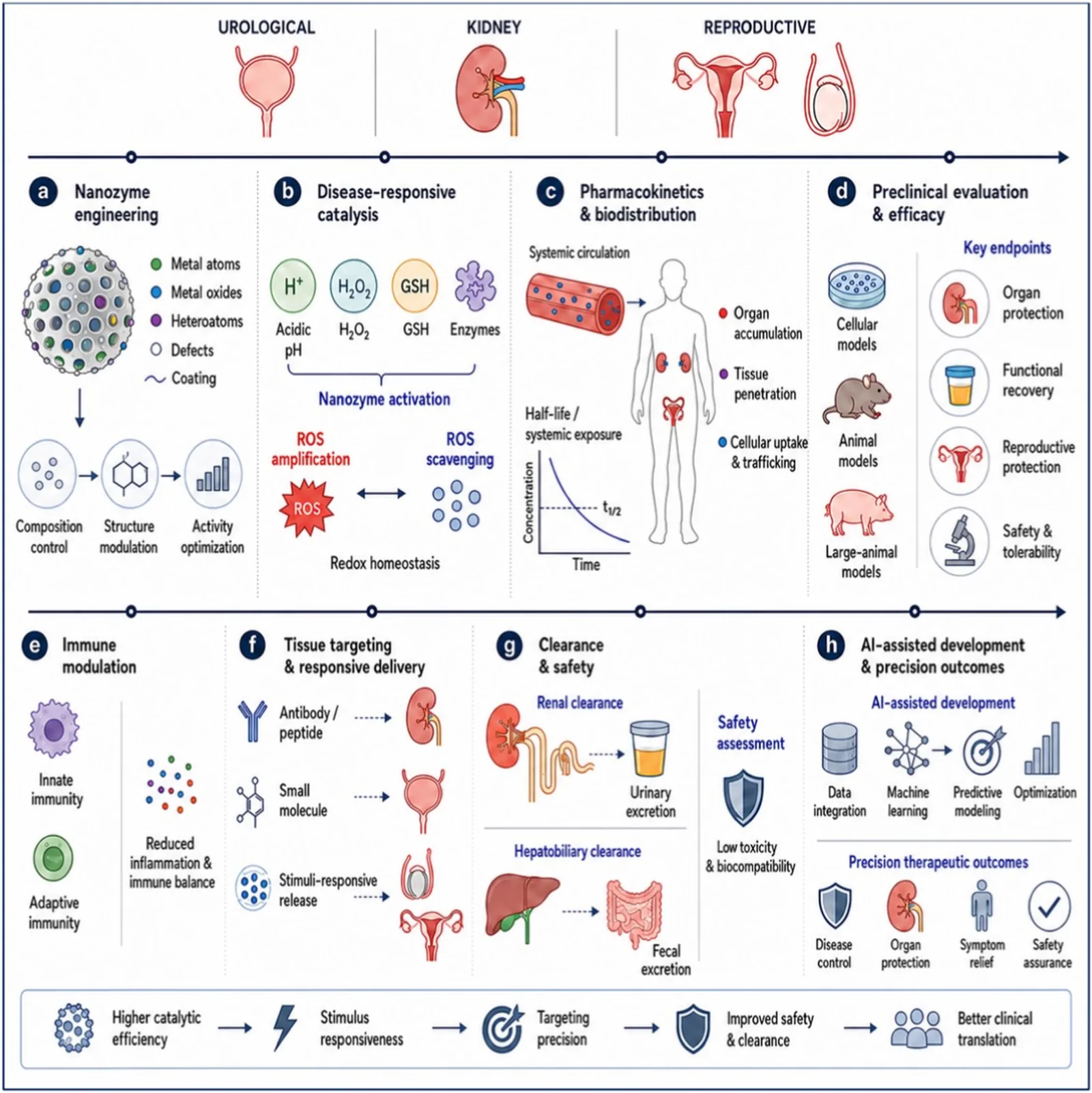
Translational framework of NCM for urological, kidney, and reproductive diseases, illustrating nanozyme engineering, structure-activity optimization, preclinical efficacy, pharmacokinetics and biodistribution, disease-responsive ROS amplification or scavenging, immune modulation, tissue targeting, renal and hepatobiliary clearance, AI-assisted development, and precision therapeutic outcomes. Image created with BioRender; concept adapted from previous studies [205,206].

For urological, kidney, and reproductive diseases, nanocatalytic systems must be carefully characterized in the preclinical context in terms of efficacy, catalytic activity, biodistribution, pharmacokinetics, and biological safety. ROS amplification and induction of ferroptosis in malignant cells, ROS scavenging, protection of mitochondria, anti-inflammatory activity, and inhibition of ferroptosis in injured renal and reproductive tissues should be evaluated *in vitro* to understand disease-specific mechanisms. Disease-relevant models should include renal tubular and glomerular cells, podocytes, urothelial and reproductive cells, relevant disease cell lines, three-dimensional (3D) spheroids, organoids, and organ-on-chip systems. Nanozyme engineering, preclinical evaluation, disease-responsive catalytic activity, and precision-based therapeutic outcomes are manifested in the translational pathway (Fig. 6) [161,205,206].

Physiologically relevant information regarding tissue penetration, cellular trafficking, catalytic activity, and response to therapy can be obtained from patient-derived organoids, xenografts, orthotopic tumor models, and animal models of nephritis, nephrotic syndrome, acute kidney injury, chronic kidney disease, ischemia-reperfusion injury, and reproductive disorders. Before clinical use, biodistribution, organelle targeting, renal handling, interactions with the immune system, and organ-specific toxicity should be assessed. Disease heterogeneity, the immune system, metabolic status, and redox status are important factors that can significantly influence therapeutic responses. Hence, the reproducibility and predictive power of models in the preclinical stage strongly rely on the use of advanced preclinical models and AI-driven structure-activity relationships [207].

### 6.2 Pharmacokinetics and biodistribution

The therapeutic effects, safety, and clinical applications of catalytic nanozymes are associated with their pharmacokinetic and biodistribution properties. The size, shape, surface charge, composition, biodegradability, catalytic stability, and functionalization of the surface affect the circulation time, tissue penetration, cellular uptake, renal filtration, and hepatobiliary clearance of nanomaterials. In the kidneys, urinary tract, or reproductive organs, biomimetic coatings, polymers, antibodies, peptides, and tissue-targeting ligands can enhance lesion targeting and minimize nonspecific tissue deposition [208].

Finally, tumor-directed platforms can take advantage of the greater penetrability of tumor vessels, receptor specificity, and sensitivity to the pathological microenvironment to optimize the delivery of drugs to renal, bladder, prostate, ovarian, cervical, and endometrial cancers. For kidney diseases, nanomaterial design should include factors related to glomerular filtration and tubular reabsorption, enabling access to podocytes, uptake by renal macrophages, and retention in inflamed or fibrotic tissue. The main steps in the process of penetration into tissues, intracellular trafficking, organelle localization, and renal or hepatobiliary clearance are summarized in Fig. 6 [209].

Controlled tissue retention followed by efficient systemic elimination of these agents from organs may be facilitated by the use of biodegradable and metabolically responsive nanoplatforms. However, long-term use of nanozymes might lead to the deposition of persistent metallic nanoparticles in the kidneys, liver, spleen, or lungs, causing changes in the function of the kidneys and their filtration capacity. Hence, there is a need to optimize the dosage, route, catalytic persistence, degradation products, and elimination pathways to ensure the safe translation of NCM across urological, kidney, and reproductive diseases [208].

### 6.3 Biosafety and Toxicological considerations

A thorough biosafety analysis is necessary because catalytic nanomaterials can affect the redox balance in healthy and diseased tissues. Pro-oxidant nanozymes may cause genotoxicity, inflammation, mitochondrial dysfunction, and oxidative injury in normal renal, urinary, and reproductive tissues of the body. However, excessive antioxidant activity or prolonged exposure to antioxidant agents can disrupt physiological redox signaling, immune defense, and normal cellular adaptation [20].

Some potential chronic toxicities of metallic nanozymes are related to their long-term presence, liberation of catalytic ions, and degradation products. The kidneys are among the organs most susceptible to the effects of nanoparticles because of their role in filtering and eliminating them. Nephrotoxicity may include glomerular damage, podocyte dysfunction, accumulation of nanoparticles in the tubules, oxidative stress, and renal dysfunction. Reproductive toxicity, immunotoxicity, inflammatory cytokine release, endocrine disruption, and fertility effects should also be evaluated [88].

The use of biomimetic engineering, biodegradable materials, disease sensor-controlled catalytic switching, and stimuli-responsive activation can minimize the off-target effects of these systems. AI-assisted design may also be used to predict toxicity, optimize the dose of nanozymes, and create a safe-by-design nanozyme. Successful translation also requires therapeutic efficacy, preservation of renal and reproductive functions, absence of significant systemic toxicity, controlled immune responses, and predictable long-term clearance [210].

### 6.4 Manufacturing and regulatory challenges

Scalable and reproducible manufacturing remains a major challenge in the clinical translation of NCMs. For clinical applications, nanozymes should have uniform particle size, shape, surface chemistry, composition, catalytic activity, defect structure, purity, stability, and biological activity. Moderate changes in these properties can alter ROS-regulating activity, tissue distribution, degradation, toxicity, and therapeutic reproducibility. Therefore, stringent quality control methods and structure-activity relationships are required [211].

Designing multifunctional systems, in which catalytic materials, targeting ligands, imaging agents, therapeutic cargoes, and biomimetic coatings are integrated with the introduction of external activation, is particularly challenging for regulatory assessment. Standardized methods are required to determine enzyme-like activity, catalytic parameters, substrate specificity, ROS generation/scavenging, degradation products, immunogenicity, reproductive toxicity, nephrotoxicity, pharmacokinetics, and batch-to-batch consistency [61].

Few long-term clinical studies and harmonized regulatory processes are available for nanozymes. For translation to urology, nephrology, and reproductive medicine, production technologies need to be scalable, characterization needs to be standardized, GMP compliance must be met, and clinically relevant potency assays and internationally agreed regulatory guidance are required [212].

### 6.5 Current clinical landscape and translational opportunities

Nanocatalytic systems used for the treatment of urological, kidney, and reproductive diseases are mostly in the preclinical phase. The potential for more widespread biomedical and cancer applications of iron oxide nanoparticles, catalytic phototherapeutic platforms, antioxidant nanozymes, biomimetic systems, and multifunctional theranostic nanomaterials is being investigated. However, direct clinical evidence for the use of nanomedicine in disease-specific therapy for nephritis, nephrotic syndrome, acute kidney injury, and reproductive disorders is limited. Thus, translational research should focus on clinically relevant models, disease-specific biomarkers, standardized safety evaluations, and clearly defined therapeutic indications [213].

The clinical translation of nanozymes could be accelerated by biomimetic engineering, AI-guided nanozyme optimization, patient-derived organoids, kidney-on-chip models, multi-omics-based patient stratification, and imaging-guided treatment. In malignant diseases, intelligent systems can amplify ROS, induce ferroptosis, activate antitumor immunity, and enable localized multimodal therapy. Catalytic systems can be used in the kidney and in non-malignant diseases of the reproductive tract to scavenge ROS, protect mitochondria, control inflammation, and provide anti-fibrotic therapy [188].

Comprehensive toxicity testing, pharmacokinetic/clearance testing, dose optimization, staged clinical trials, real-time response monitoring, and integration into personalized therapeutic decision-making should be incorporated into future clinical practice. To translate preclinical nanocatalytic platforms into safe and effective precision therapeutics for urological, kidney, and reproductive diseases, industrial investment, computational engineering, regulatory harmonization, and interdisciplinary collaboration are required [214].

## 7 Future perspectives

### 7.1 Next-generation catalytic nanozymes

**Fig. 7:**
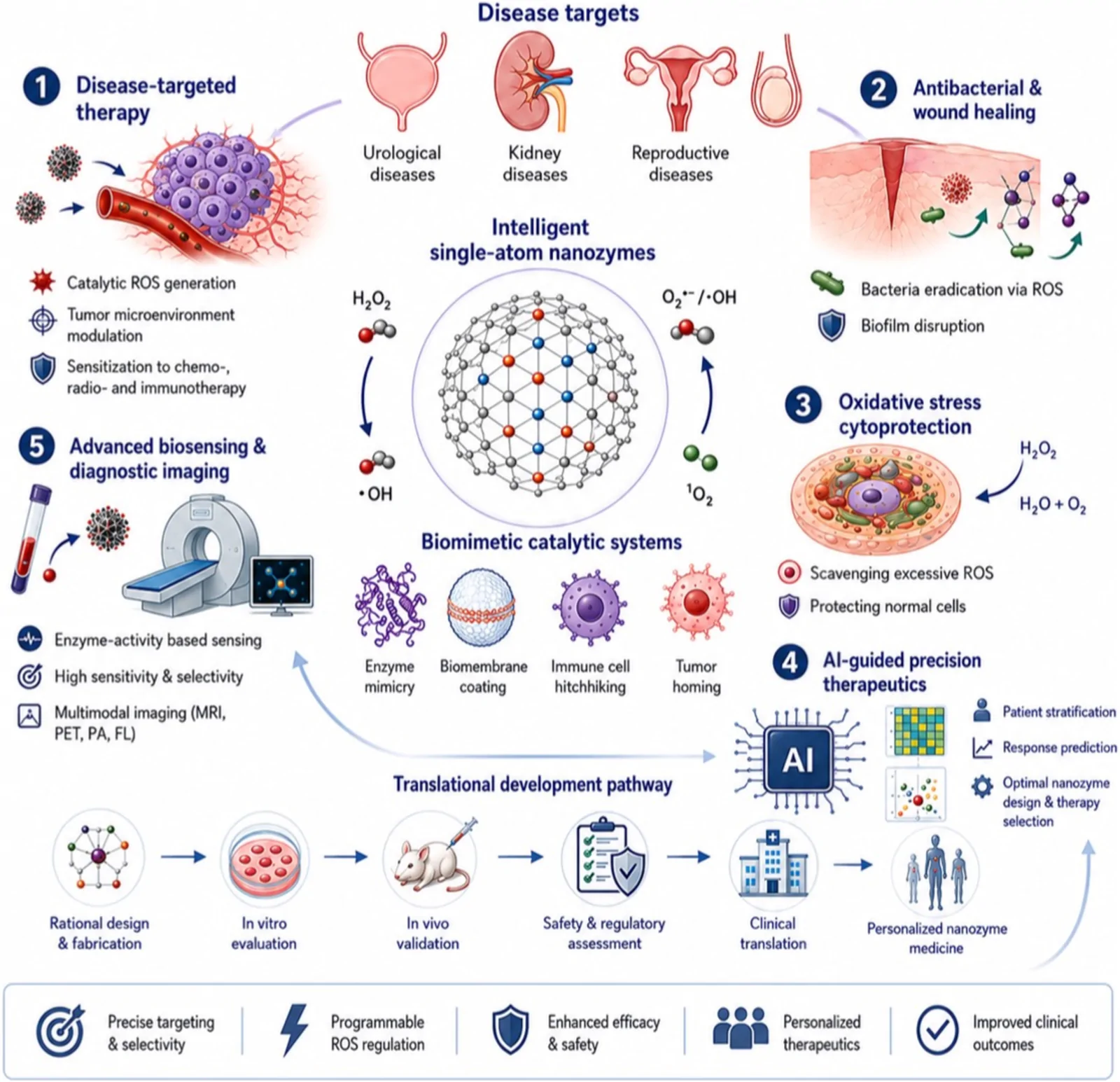
Intelligent single-atom nanozymes for precision therapy and diagnosis of urological, kidney, and reproductive diseases. These biomimetic catalytic systems enable targeted ROS regulation, antibacterial and wound-healing activity, oxidative stress protection, multimodal biosensing, and AI-guided therapeutic optimization, supporting personalized clinical translation. Image created with BioRender. Concept adapted from previous studies [216].

Next-generation catalytic nanozymes are antipiciated to have greater catalytic efficiency, biological adaptability, and therapeutic precision in urological, kidney, and reproductive diseases. The activity of nanozymes, including their catalytic specificity, tissue targeting, and biocompatibility, can be enhanced by optimizing their composition and surface functionalization, and by engineering structural defects and single-atom catalytic centers. Multi-enzyme-mimetic systems consisting of oxidase, peroxidase, CAT, SOD, and GPX-like activities could allow more context-dependent and precise control of ROS than single-function nanozymes. Future directions for nanozymes include intelligent single-atom nanozymes, biomimetic catalytic systems, precision therapeutics for clinical use guided by AI, and clinically oriented translational frameworks (Fig. 7) [215,216].

As reported by Liu, the potential for cascade reactions, controlled drug release, adaptive modulation of ROS, and feedback-responsive activation of self-regulating catalytic systems could provide an additional level of therapeutic efficacy and safety. In nonmalignant conditions, these systems should instead eliminate excess ROS, protect mitochondria, reduce inflammation, and minimize fibrosis. These strategies include responsive polymers, biomembrane coatings, immune cell hitchhiking, and tissue-homing nanozymes, which can improve stability in circulation, cargo delivery to the interior of cells, renal or reproductive tissue localization, and catalytic activation of nanozymes in a pathological context [157].

### 7.2 Personalized and precision nanocatalytic therapeutics

To treat urological, kidney, and reproductive diseases with molecular and pathological diversity, personalized nanocatalytic therapy is necessary. These disorders differ substantially in terms of genetic and redox alterations, metabolic pathways, immune composition, fibrosis, tissue injury, and therapeutic responsiveness. Multi-omics analyses of genomics, transcriptomics, proteomics, metabolomics, epigenomics, and computational biomarkers can help identify appropriate molecular targets, forecast catalytic reactions, and help choose pro-oxidant or antioxidant systems for nanozymes [217].

AI methods, including machine learning and neural networks, can support therapeutic modeling, patient stratification, dose optimization, toxicity prediction, monitoring, and response-prediction. These strategies might enable the matching of ROS-enhancing nanozymes to cancer cells and identify patients with kidney or reproductive damage who may benefit from catalytic antioxidant, anti-inflammatory, or antifibrotic treatments. AI-driven precision nanocatalytic therapy offers several advantages, including enhanced efficacy, minimal systemic toxicity, maintenance of kidney and reproductive functions, and more predictable and long-term clinical outcomes [218].

The future clinical translation of precision nanocatalytic therapeutics should focus on disease-specific evaluations across major GU malignancies. Early phase clinical studies should investigate biomarker-guided catalytic nanozyme platforms in ovarian, bladder, renal cell, prostate, cervical, endometrial, and upper tract urothelial cancers, while integrating molecular profiling, multimodal imaging, and adaptive trial designs for patient stratification. Such precision oncology strategies will facilitate individualized catalytic therapy, optimize therapeutic efficacy, and accelerate the clinical translation of NCM across diverse GU malignancies.

### 7.3 Biomimetic and living catalytic nanomedicine

Biomimetic and living catalytic nanomedicine are emerging approaches for enhancing biocompatibility, tissue targeting, and adaptive therapeutic activity in urological, kidney, and reproductive diseases. Immune evasion, circulation stability, intracellular delivery, and selective accumulation in tumors, inflamed kidneys, urinary tissues, and reproductive organs can be enhanced by cell membrane-coated nanozymes, exosome-inspired platforms, immune cell-mediated delivery systems, and biohybrid catalytic therapeutics. These systems may also modulate ROS, inflammation, metabolism, and immune responses in the tumor microenvironment [177].

The potential applications of catalytic nanomedicine can be further extended by the design of synthetic enzymes, programmable biomaterials, catalytic artificial centers, bacteria, and living cells. These systems allow for dynamic metabolic regulation, immune modulation, oxidative cytoprotection, repair, and adaptive therapeutic activation. In cancer, living catalytic platforms may promote localized tumor destruction and immune activation. In the kidneys and in non-malignant reproductive disorders, they may provide antioxidant and anti-inflammatory protection and support tissue regeneration. Thus, there is great potential to gain insights from biomimetic and living systems to enhance the therapeutic specificity and clinical translation.

### 7.4 Emerging opportunities in clinical translation

The clinical application of NCM can open up new possibilities for the precision treatment of urological, kidney, and reproductive diseases. Catalytic systems can be combined with CDT, immunotherapy, phototherapy, ferroptosis induction, gene therapy, and metabolic interventions to improve their therapeutic efficacy. Nanozymes can be combined with anti-inflammatory, antioxidative, antifibrotic, immunomodulatory, and regenerative therapeutic agents for the treatment of nephritis, nephrotic syndrome, acute kidney injury, chronic kidney disease, and inflammatory reproductive disorders. Intelligent theranostic systems, which can combine biosensing, targeted delivery, catalytic activation, multimodal treatment, and imaging guidance, can enhance therapeutic precision and provide real-time patient monitoring [213].

The hurdles to clinical adoption include regulatory harmonization, scalable production, validated potency testing, and exhaustive safety testing. The reproducibility of the synthesis, structure–activity characterization, standard catalytic measurements, pharmacokinetic studies, nephrotoxicity and reproductive toxicity testing, and long-term monitoring are crucial for maintaining the therapeutic consistency and biosafety. Rational nanozyme design and disease-specific *in vitro* and *in vivo* validation should be followed by organoid testing, pharmacological optimization, clinical trials, and treatment personalization (Fig. 7). These platforms must be clinically applied through collaboration and interdisciplinary exchange of knowledge and experience among urology, nephrology, reproductive medicine, nanotechnology, computational biology, pharmaceutical science, and AI [211].

## 8 Conclusion

NCM provides a flexible approach for the precision treatment of urological, renal, and reproductive diseases by regulating catalytic therapeutic reactions and oxidative stress on a disease-specific basis. In recent years, several types of biocatalytic nanomaterials, such as metallic and metal-oxide nanozymes, hybrid nanozymes, biomimetic platforms, and stimuli-responsive materials, have been developed to enhance the catalytic activity of nanomaterials and make them more tissue-selective and adaptable for therapeutic uses. In malignant diseases, these platforms can amplify intratumoral ROS, induce ferroptosis and metabolic collapse, and activate antitumor immunity by exploiting acidic pH, hypoxia, and elevated H_2_O_2_ levels, while depleting or disabling GSH-dependent antioxidant defenses within the TME. Nanozymes serve as antioxidants to eliminate excessive ROS, maintain mitochondrial function, inhibit inflammation, and reduce fibrosis in nephritis, nephrotic syndrome, acute kidney injury, chronic kidney disease, ischemia-reperfusion injury, and nonmalignant reproductive disorders.

The basis of NCM is the bidirectional regulation of ROS. Pro-oxidant systems may lead to mitochondrial dysfunction, lipid peroxidation, DNA damage, ferroptotic or apoptotic cell death, immunogenic tumor destruction, and remodeling of the TME. In contrast, SOD-, CAT-, and GPx-like nanozymes can reverse redox imbalance, prevent damage to the kidneys and reproductive organs, and reduce inflammation. Precision oncology has expanded to include therapeutic strategies that combine CDT, catalytic phototherapy, metabolic regulation, immunocatalytic treatment, targeted drug delivery, and multimodal theranostics using NCM. Patient stratification and subsequent matching of therapies are further enhanced by the use of multi-omics profiling, biomarker-guided targeting, organoid screening, imaging-guided therapy, and the design of nanozymes with the aid of AI.

Despite their distinct tissue origins, major GU malignancies, including ovarian, bladder, renal cell, prostate, cervical, endometrial, and upper tract urothelial cancers, share conserved redox vulnerabilities, including acidic extracellular pH, elevated H_2_O_2_ levels, GSH-mediated antioxidant defenses, hypoxia, and immunosuppressive tumor microenvironments, providing a common mechanistic foundation for nanocatalytic therapy in GU cancers. Nevertheless, successful clinical translation will require disease-specific precision strategies that integrate catalytic ROS modulation with molecular targeting, such as PSMA-directed delivery in prostate cancer, HIF-associated approaches in RCC, intravesical nanozyme administration for bladder cancer, and CA125-or folate receptor-targeted platforms in ovarian cancer.

Although significant preclinical advances have been made, clinical translation remains limited by an incomplete understanding of pharmacokinetics, biodistribution, catalytic persistence, and long-term toxicity; inadequate renal and reproductive safety data; poor manufacturing reproducibility; and unresolved regulatory requirements. Future studies should focus on the development of nanozymes with greater biodegradability and lower organ-specific toxicity, standardization of their catalytic potency, creation of clinically relevant disease models, scaling up of production, and thorough investigation of their degradation products. Ongoing developments in biomimetic engineering, computational design, precision diagnostics, and translational pharmacology will open the door for a new generation of personalized therapeutics for urological, kidney, and reproductive diseases based on advanced biocatalytic nanomaterials.

## CRediT authorship contribution statement

**Huimin Li:** Investigation, Methodology, Writing – review & editing. **Ye Geng:** Investigation, Methodology, Writing – review & editing. **Lina Wang:** Methodology, Writing – original draft, Writing – review & editing. **Shangxu Jiang:** Data curation, Investigation, Writing – original draft. **Ming Du:** Conceptualization, Writing – original draft, Writing – review & editing. **Yanan Sun:** Conceptualization, Writing – original draft, Writing – review & editing. **Li Li:** Conceptualization, Supervision, Writing – review & editing. **Xinwang Zhu:** Conceptualization, Methodology, Writing – original draft, Writing – review & editing.

## Funding declaration

Not applicable.

## Declaration of competing interests

The authors declare that they have no known competing financial interests or personal relationships that could have appeared to influence the work reported in this paper.

## Data Availability

No data was used for the research described in the article.
